# Synthesis of mesoporous salen polymer nanospheres for highly efficient photocatalytic extraction of uranium from seawater

**DOI:** 10.1093/nsr/nwag569

**Published:** 2026-09-03

**Authors:** Yenpin Chu, Yan Ai, Yuxuan Li, Jun Wang, Kailin Li, Ziqing Yin, Xiaomin Li, Tongtao Li, Dongyuan Zhao, Wei Li

**Affiliations:** Department of Chemistry, Laboratory of Advanced Materials, Shanghai Key Laboratory of Molecular Catalysis and Innovative Materials, State Key Laboratory of Porous Materials for Separation and Conversion, Fudan University, Shanghai 200433, China; Department of Chemistry, Laboratory of Advanced Materials, Shanghai Key Laboratory of Molecular Catalysis and Innovative Materials, State Key Laboratory of Porous Materials for Separation and Conversion, Fudan University, Shanghai 200433, China; Department of Chemistry, Laboratory of Advanced Materials, Shanghai Key Laboratory of Molecular Catalysis and Innovative Materials, State Key Laboratory of Porous Materials for Separation and Conversion, Fudan University, Shanghai 200433, China; Department of Chemistry, Laboratory of Advanced Materials, Shanghai Key Laboratory of Molecular Catalysis and Innovative Materials, State Key Laboratory of Porous Materials for Separation and Conversion, Fudan University, Shanghai 200433, China; Department of Chemistry, Laboratory of Advanced Materials, Shanghai Key Laboratory of Molecular Catalysis and Innovative Materials, State Key Laboratory of Porous Materials for Separation and Conversion, Fudan University, Shanghai 200433, China; Department of Chemistry, Laboratory of Advanced Materials, Shanghai Key Laboratory of Molecular Catalysis and Innovative Materials, State Key Laboratory of Porous Materials for Separation and Conversion, Fudan University, Shanghai 200433, China; Department of Chemistry, Laboratory of Advanced Materials, Shanghai Key Laboratory of Molecular Catalysis and Innovative Materials, State Key Laboratory of Porous Materials for Separation and Conversion, Fudan University, Shanghai 200433, China; Department of Chemistry, Laboratory of Advanced Materials, Shanghai Key Laboratory of Molecular Catalysis and Innovative Materials, State Key Laboratory of Porous Materials for Separation and Conversion, Fudan University, Shanghai 200433, China; Department of Chemistry, Laboratory of Advanced Materials, Shanghai Key Laboratory of Molecular Catalysis and Innovative Materials, State Key Laboratory of Porous Materials for Separation and Conversion, Fudan University, Shanghai 200433, China; Department of Chemistry, Laboratory of Advanced Materials, Shanghai Key Laboratory of Molecular Catalysis and Innovative Materials, State Key Laboratory of Porous Materials for Separation and Conversion, Fudan University, Shanghai 200433, China

**Keywords:** mesoporous salen polymers, photocatalytic uranium extraction, self-assembly

## Abstract

Efficient uranium extraction from seawater is essential to the long-term sustainability of nuclear energy, yet the rational design of high-performance materials for this purpose remains a formidable challenge. Herein, we develop an ionization-regulated self-assembly strategy to synthesize mesoporous salen polymer nanospheres (mDSR) for photocatalytic uranium extraction. Partial ionization improves the aqueous solubility of otherwise insoluble salen ligands and enhances their reactivity toward formaldehyde, while preserving sufficient precursor–micelle interactions for co-assembly, thereby directing the formation of ordered mesostructures. This strategy is facile, universal, and it can accommodate a series of structurally distinct salen ligands, enabling the modular construction of mesoporous polymers with programmable molecular functions. The mDSR nanospheres feature a large pore size (6.2 nm), a high surface area (68 m^2^ g^−1^) and intrinsic photocatalytic activity. As a result, under simulated sunlight and without sacrificial agents, mDSR achieves a high uranium uptake capacity of 867 mg U g^−1^ from a 10 ppm U(VI) solution, outperforming most adsorbents. This protocol establishes a new paradigm for functional mesoporous polymers.

## Teaser

This study constructs mesoporous salen polymers that integrate large mesopores, plentiful adsorption and photocatalytic sites, thereby enabling high‑performance photocatalytic uranium extraction from seawater.

## INTRODUCTION

Nuclear power is a key technology for sustained, large-scale low-carbon electricity generation, and its long-term development depends on a secure and sustainable uranium supply [1–4]. Seawater contains ∼99.8% of the global uranium inventory and thus represents a vast resource capable of supporting the long-term expansion of nuclear energy [5–9]. However, the ultra-low concentration of uranium (∼3.3 ppb), competition from many interfering ions, high salinity and the complexity of the marine environment make uranium extraction from seawater exceptionally challenging [10–15]. Photocatalytic uranium extraction has emerged as a promising strategy, as it can enhance uranyl binding at coordination sites through photothermal effects and mitigate biofouling via photoinduced reactive oxygen species [16–20]. Accordingly, crystalline porous materials such as metal-organic frameworks and covalent organic frameworks have emerged as promising platforms for photocatalytic uranium extraction because of their well-defined pore structures, abundant active sites and tunable optoelectronic properties [21–24]. However, many of these materials are predominantly microporous, which restricts the diffusion of uranyl-based complexes with large kinetic diameters (0.59–1.15 nm). Moreover, deposited uranium products would block the pores and cover internal active sites, slowing extraction and decreasing site utilization [11,25].

Mesoporous materials generally possess large pore sizes and high surface areas [26–29], which can facilitate the rapid transport and adsorption of uranium species [30–34]. Meanwhile, their large pore volumes can also accommodate solid uranium products generated during photocatalysis, thereby alleviating pore blockage [33,35]. Over the past decades, mesoporous materials have evolved from silica-based solids into a broad family encompassing metal oxides, metals, carbons and organic polymers [36–41]. Despite this compositional diversity, most mesoporous materials lack the molecularly defined binding sites and tunable photoactive units required to combine selective uranium recognition with efficient photochemical conversion.

Here, we use salen ligands as molecular building blocks to construct ordered mesoporous polymer nanospheres (mDSR) through ionization-regulated self-assembly, thereby integrating uranyl coordination and photocatalytic functionality within a single framework. Tuning the ionization degree of precursors renders the otherwise insoluble salen ligands water soluble, allowing them to undergo polymerization with formaldehyde while co-assembling with F127 monomicelles into ordered mesostructures. Furthermore, the generality of this strategy allows the incorporation of structurally diverse salen ligands, providing access to mesoporous polymers with tailored functionalities. The mDSR exhibits exceptionally rapid adsorption kinetics and efficient photocatalytic uranium extraction, achieving uranium uptake capacities of 867 mg U g^−1^ from a 10 ppm U(VI) solution and 5715 mg U g^−1^ from an 80 ppm U(VI) solution after 10 h of light irradiation, outperforming conventional adsorbents. Our work paves a new way to design and synthesize next-generation porous polymers for various applications.

## RESULTS AND DISCUSSION

The mDSR was synthesized by the ionization-regulated self-assembly strategy as illustrated in Fig. 1. The field-emission scanning electron microscopy (FESEM) image reveals that the mDSR nanospheres possess uniform spherical morphology with an average diameter of ∼220 nm (Fig. 2a). At higher magnification, well-defined and highly ordered mesopores are clearly visible across the nanosphere surfaces (Fig 2b). Transmission electron microscopy (TEM) images (Fig. 2c and d) further show cylindrically arranged mesoporous channels with a diameter of ∼6 nm. The small-angle X-ray scattering (SAXS) pattern (Fig. 2f) exhibits three well-resolved scattering peaks at *q* values of 0.035, 0.060 and 0.070 Å^−1^, which can be indexed to the (100), (110) and (200) scattering reflections of p6mm mesostructures. The N_2_ sorption isotherms of the mDSR display a representative type-IV curve. The Brunauer–Emmett–Teller (BET) surface area can be calculated to be 68 m^2^ g^−1^ (Fig. 2g). The pore size distribution is very narrow and centered at ∼6.2 nm (Fig. 2g), in good agreement with the TEM result (Fig. 2d). High-angle annular dark-field scanning TEM (HAADF-STEM) image and the corresponding energy dispersive X-ray (EDX) element mapping images (Fig. 2e) confirm the homogeneous distribution of C, N and O throughout the polymer framework.

**Figure 1.**
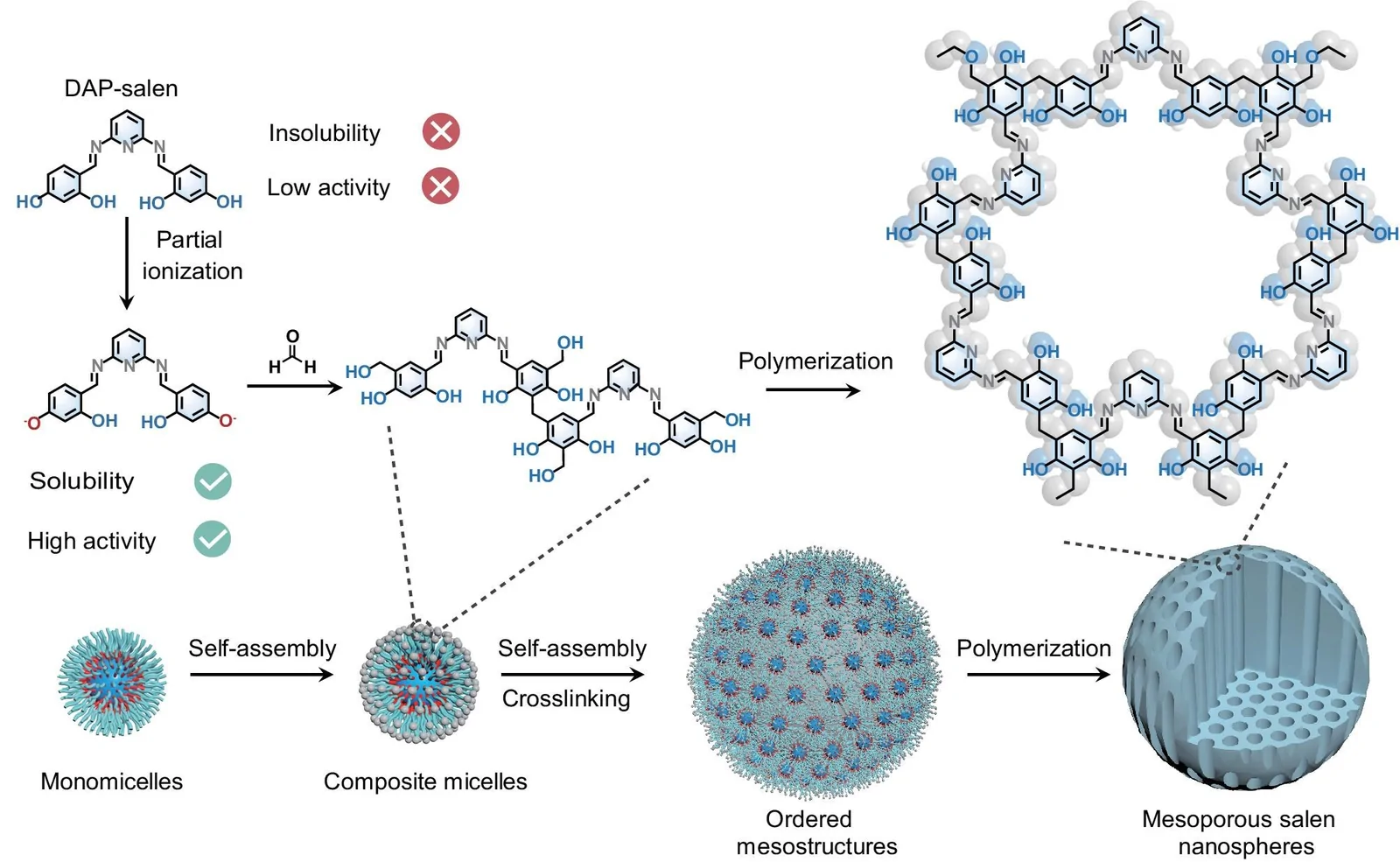
Schematic illustration of the ionization-regulated self-assembly strategy for mesoporous salen polymer nanospheres.

**Figure 2.**
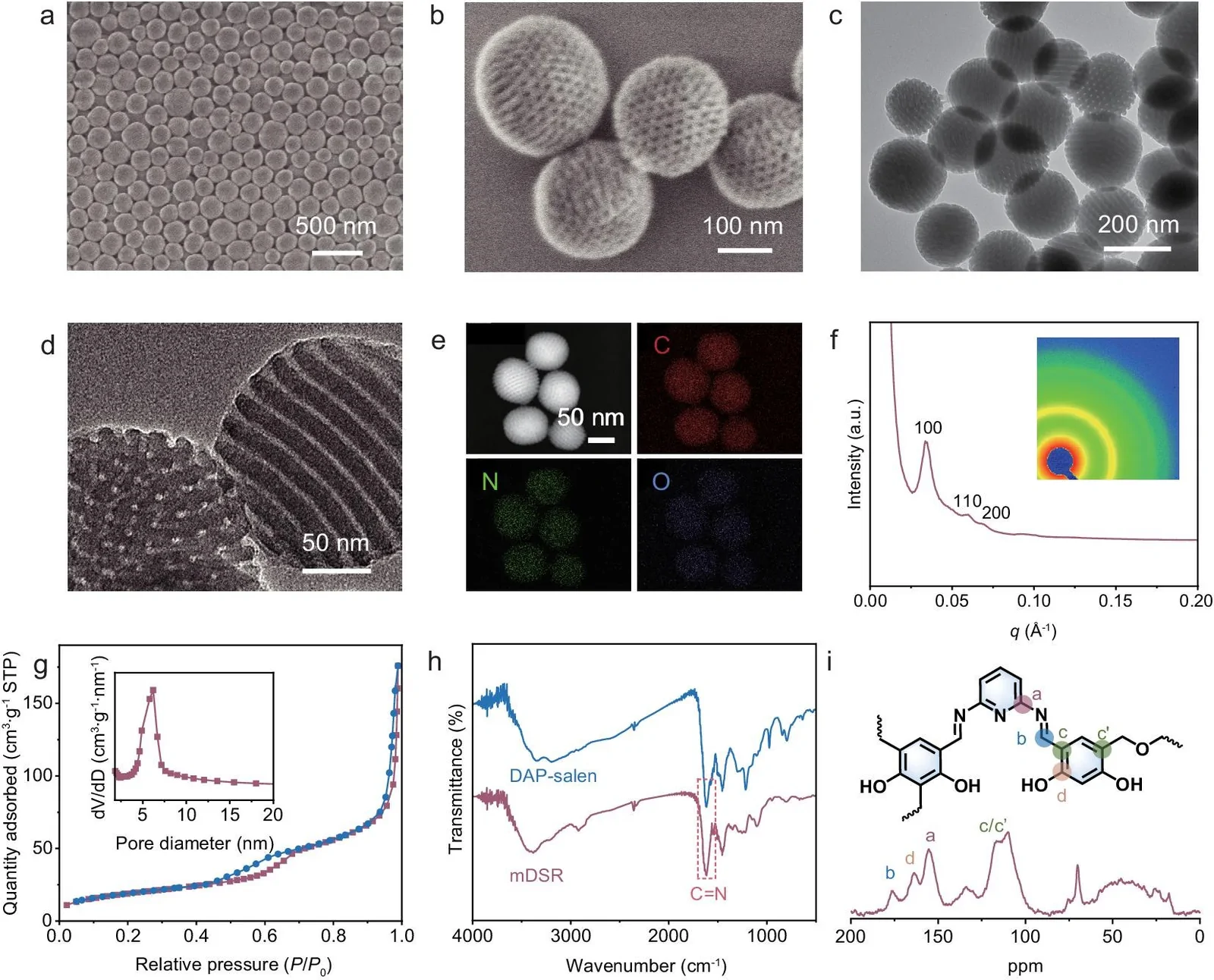
(a and b) FESEM images, (c and d) TEM images, (e) HAADF-STEM and corresponding EDX elemental mapping images, (f) SAXS pattern, (g) N_2_ isotherms and corresponding pore-size distribution (inset), (h) FT-IR spectrum and (i) solid-state ^13^C CP/MAS NMR spectrum of mDSR.

The high-resolution X-ray photoelectron spectroscopy (XPS) spectrum of the C 1s region (Fig. S1b) exhibits a peak at 285.9 eV, ascribed to C–N bonds. The N 1s region (Fig. S1c) can be deconvoluted into three peaks, assigned to C=N (398.6 eV), C–N (400.0 eV), NH_4_^+^/NR_4_^+^ (401.5 eV). These XPS results further confirm the presence of chemically distinct nitrogen-containing functionalities on the mDSR surface. The Fourier-transform infrared (FT-IR) spectra show the same characteristic C=N stretching band at 1616 cm^−1^ in both mDSR and DAP-salen, indicating the preservation of the salen framework after polymerization (Fig. 2h) [42]. Meanwhile, new absorption bands at 2921 and 2850 cm^−1^ corresponding to aliphatic C–H stretching vibrations emerge in mDSR (Fig. 2h), supporting the formation of methylene linkages through formaldehyde-mediated polycondensation [43]. The ^13^C cross polarization/magic angle spinning nuclear magnetic resonance (CP/MAS NMR) spectrum of mDSR shows peaks corresponding to the –C=N, –C–OH moiety and pyridine ring at 176 ppm (b) [42], 168 ppm (d) and 155 ppm (a) [43] (Fig. 2i). These spectroscopic results confirm the successful incorporation of salen-derived coordination motifs, including pyridine nitrogen and phenolic hydroxyl groups, into the mesoporous polymer framework. In addition, the synthesis can be easily scaled up (Fig. S2).

To reveal the formation mechanism of the mDSR, the effect of ionization degree was first investigated by varying the solution pH value (Fig. 3a). With increasing pH, the UV–vis spectra exhibit a progressive red shift and a pronounced increase in absorbance, indicating that the phenolic hydroxyl groups of DAP-salen are deprotonated to form phenolate anions with stronger electron-donating ability by the rising pH value and the precursor solubility in solution is substantially enhanced by the ionization (Fig. 3b) [44,45]. ^1^H NMR spectra reveal pH-dependent changes in the interactions between the ionized precursors and water molecules (Fig. 3c). Phenolic hydroxyl protons of ionized DAP-salen form hydrogen bonds with water oxygen atoms, which enhance the shielding effect of the water oxygen atoms on the adjacent protons and shift the corresponding signals to high field. In contrast, phenolate anions of ionized DAP-salen form hydrogen bonds with water hydrogen atoms, which reduce the shielding effect and cause a downfield shift. These results indicate that the appropriate ionization effectively enhances the aqueous solubility of DAP-salen while establishing suitable hydrogen-bonding interactions with the template. Excessive ionization, however, changes the precursor from predominantly a hydrogen-bond donor to predominantly a hydrogen-bond acceptor, thereby preventing effective self-assembly with F127 micelles. Furthermore, the final polymers derived from ionized/non-ionized precursors were characterized by TEM. At low pH, the predominantly non-ionized DAP-salen precursors yield nonporous particles (Fig. S3a). Increasing the pH to 9.3 results in uniform nanospheres with well-defined ordered mesochannels (Fig. S3b). Upon further increasing the pH, the mesostructural order degree gradually deteriorates, and the fully ionized precursors again produce nonporous materials with poorly defined morphologies (Fig. S3a and d). Similar trends are observed when different bases are used for mDSR synthesis (Fig. S4). In the KOH system, salt precipitation can be observed in the ethanol–water medium, accompanied by a lower solution pH and insufficient precursor ionization. Consequently, the resulting materials exhibit lower morphological uniformity and mesostructural ordering than those prepared using NaOH or TMAOH. These results show that ordered mesoporosity and uniform spherical morphology can be simultaneously achieved only within an intermediate ionization window. These results also explain the critical role of NH_4_Cl in the synthesis system: the buffer system formed with NH_4_Cl and base effectively prevents drastic pH fluctuations and ensures the stability of the ionization degree. In contrast, replacing NH_4_Cl with NaCl eliminates the NH_4_^+^/NH_3_ buffering effect, causing a rapid increase in pH and ultimately yielding fish-scale-like lamellar structures (Fig. S9a and b).

**Figure 3.**
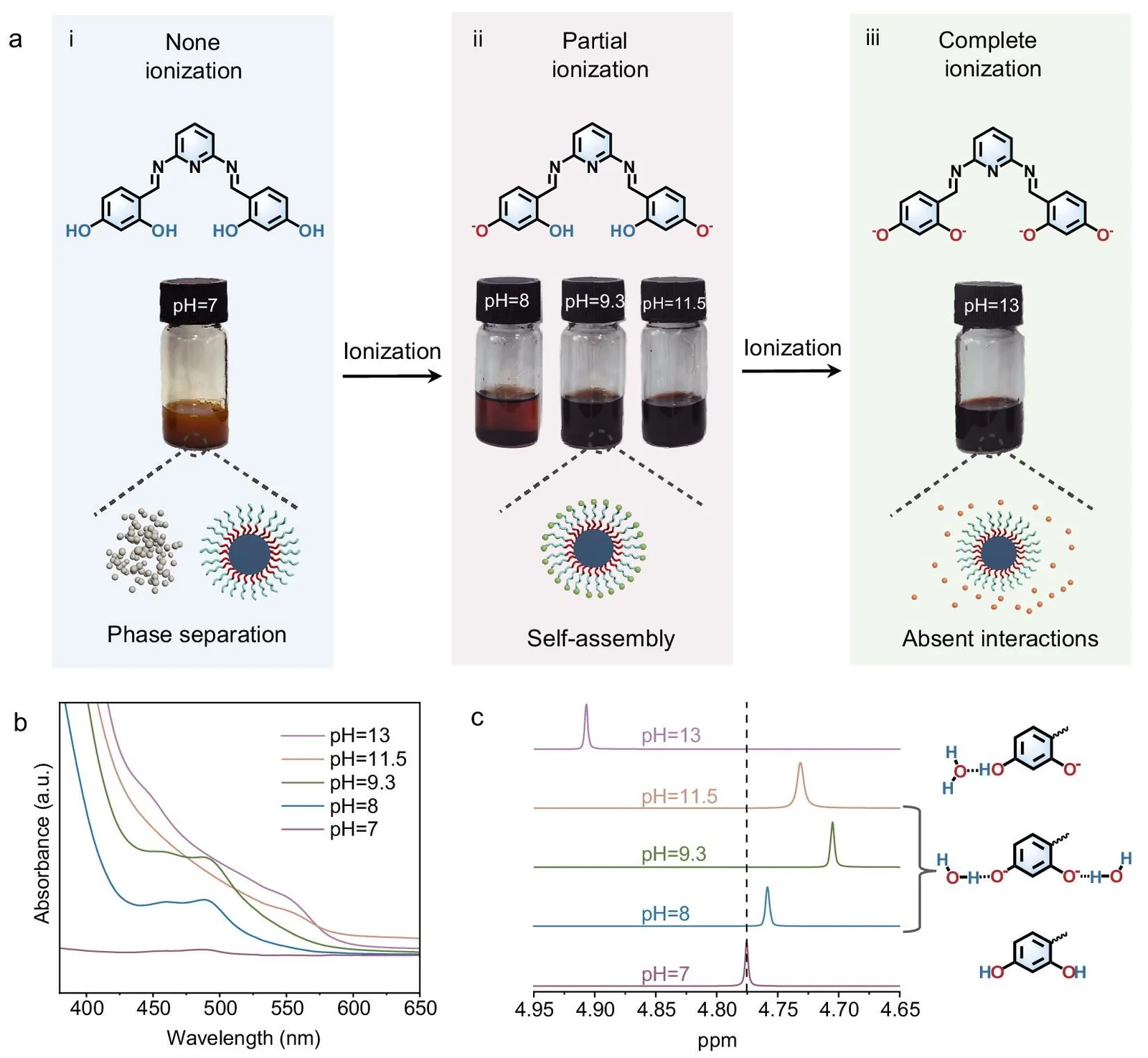
(a) Schematic illustration of the self-assembly behavior between DAP-salen and F127 micelles at different ionization degrees, and optical photographs after ionization for 3.5 h at different pH values. (b) UV–vis spectra of the filtrate obtained after ionizing DAP-salen with different pH values for 3.5 h. (c) ^1^H NMR spectra of the filtrates and corresponding schemes (all reagents are replaced with deuterated reagents).

Meanwhile, the influence of the pre-stirring time was examined. As the pre-stirring time increases, the hydroxyl stretching band shifts slightly toward higher wavenumbers, indicating an increase in overall number of hydrogen-bonding interactions (Fig. S5a). The resulting particles progressively decrease in size and subsequently evolve into irregular aggregates at prolonged pre-stirring times (Fig. S5b). Meanwhile, the mesostructural order degree gradually decreases (Fig. S6), indicating that an appropriate pre-equilibration period is required for the formation of ordered mesoporous nanospheres.

Furthermore, the effects of anions species were investigated by varying the NH_4_Cl/(NH_4_)_2_SO_4_ ratio. Typically, F108 does not form micelles in ethanol–water mixtures owing to its low packing parameter [46]. In our F108 system, increasing the sulfate fraction progressively enhances mesostructural ordering and ultimately yields a well-defined Im$\bar{3}$m mesophase (Figs S7 and S8). In contrast, the same increase in sulfate content gradually reduces the structural order of the F127-derived materials, indicating that inorganic salts can effectively regulate the packing parameter of surfactants. Moreover, anions species play a crucial role in governing the final morphology of mDSR due to their coagulating capacity (Fig. S9) [47,48]. Thus, the anions species strongly influence both mesophase selection and particle morphology.

Finally, the effects of swelling-agent content, surfactant, precursors amount and reaction temperature were investigated. Increasing the swelling-agent content progressively enlarges the mesopore size, while the pore ordering decreases to some extent (Fig. S10). With increasing F127 amount, particle aggregation is suppressed and the average particle size decreases, whereas the mesopore diameter remains nearly unchanged (Fig. S11). In addition, the mesostructure and particle morphology of the resulting materials can be effectively tuned by varying the hydrophobic/hydrophilic ratio of surfactants, as the continuous increase in hydrophobic/hydrophilic ratio can induce a gradual increase in the packing parameter accompanied with micellar structure transformation from spherical micelles to lamellar assemblies, thereby directing the formation of non-porous nanosheet-like structures [46] (Fig. S12). Variations in the precursors amount and reaction temperature further result in systematic changes in the particle size of the polymer nanospheres (Figs S13 and S14). Moreover, the crosslinking strategy also plays an important role in regulating particle size and mesostructural ordering (Fig. S15). Compared with direct addition of formaldehyde, HMTA provides a better balance between polymerization and self-assembly, resulting in larger particles with more ordered mesostructures [49].

On the basis of the above observations, an ionization-regulated self-assembly mechanism is proposed for the formation of mDSR (Figs 1 and 3a). In general, the ionization degree of DAP-salen simultaneously governs its aqueous solubility, association with F127 micelles and polymerization reactivity toward formaldehyde, giving rise to three distinct assembly modes. In the non-ionized state [Fig. 3a(i)], the strong hydrophobicity and poor aqueous solubility of DAP-salen hinder its effective association with the micelles. Therefore, the system proceeds without organized micellar co-assembly, yielding non-porous products (Fig. S3a). Upon partial ionization [Fig. 3a(ii)], the generated phenolate groups improve precursor solubility and enhance its reactivity toward formaldehyde, while the remaining phenolic hydroxyl groups retain hydrogen-bonding interactions with the hydrophilic domains of F127. This intermediate ionization state establishes an appropriate kinetic balance between precursor polycondensation and micellar self-assembly, leading to the formation of ordered mesoporous polymers (Fig. S3b and c). At a high ionization degree [Fig. 3a(iii)], the reduced fraction of phenolic hydroxyl groups weakens precursor–template interactions, whereas the enhanced precursor reactivity accelerates polymerization. The resulting kinetic mismatch suppresses ordered co-assembly and produces disordered or non-porous products (Fig. S3d). Therefore, partial ionization provides an effective means of balancing precursor solubilization, micellar association and polymerization kinetics, thereby directing the formation of ordered mesostructures.

To examine the generality of the ionization-regulated self-assembly strategy, DAP-salen was replaced with a series of structurally distinct salen and salen-type monomers, including salen, OPD-salen, Chiral-salen and DETA-salen. Using this strategy, structurally diverse salen-based precursors can be incorporated into mesoporous polymer frameworks, yielding materials with distinct particle morphologies (Fig. 4) and ordered mesostructures (Fig. S16). The corresponding ^13^C CP-MAS NMR spectra confirm the incorporation of the characteristic salen-derived motifs into the polymer frameworks (Fig. S17). This broad precursor compatibility provides a modular route to mesoporous polymers with tunable framework chemistry and particle morphology.

**Figure 4.**
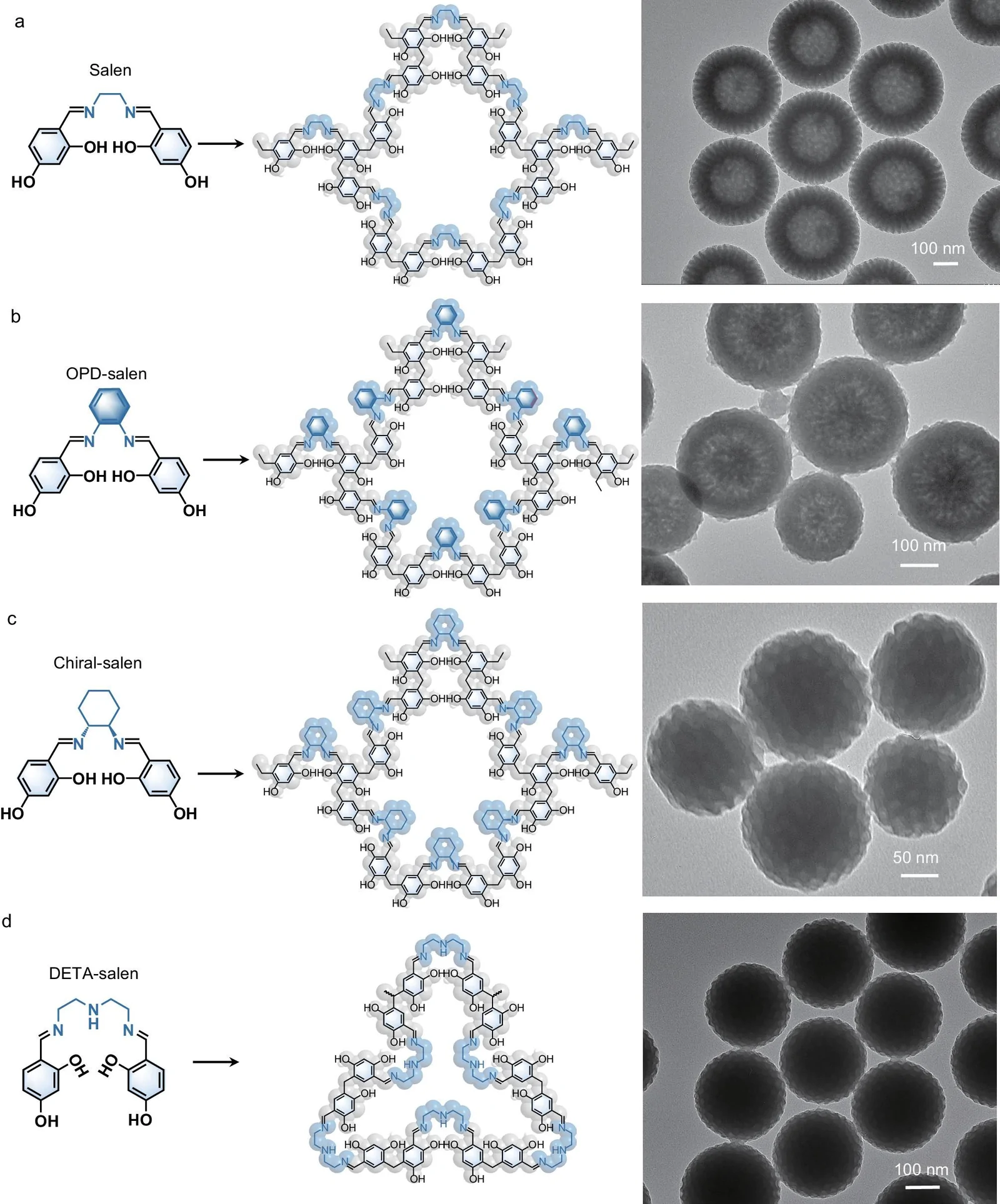
Universality of the ionization-regulated self-assembly strategy. Molecular structures, proposed polymer-network models, and TEM images of mesoporous polymer nanospheres derived from (a) salen, (b) OPD-salen, (c) Chiral-salen and (d) DETA-salen.

The uranium capture performance of mDSR was evaluated from a uranium-spiked aqueous solution under simulated sunlight. After 10 h, mDSR achieves uranium uptake capacities of 867 mg U g^−1^ from a 10 ppm uranyl(VI) solution and 5715 mg U g^−1^ from an 80 ppm uranyl(VI) solution, respectively (Fig. 5a). In the dark, mDSR reaches adsorption equilibrium within only 30 min, demonstrating rapid adsorption kinetics (Fig. S18). However, the equilibrium uptake in the dark is only 32 mg U g^−1^, substantially lower than that observed under light irradiation, indicating a pronounced photocatalytic contribution to uranium extraction. For comparison, mesoporous resorcinol–formaldehyde polymer (mRF) and mesoporous polydopamine (mPDA) were used as control materials, and their uranium-extraction performances were also evaluated under identical conditions (Fig. S19). mPDA and mRF exhibit uranium uptake capacities of 577 and 510 mg U g^−1^, respectively (Fig. 5b). Because natural seawater is weakly alkaline and contains numerous competing ions, including vanadium-containing species [11,50], the effects of pH and ion competition were further examined (Fig. 5c). Although the highest uranium uptake is obtained at pH = 6, mDSR retains a high capacity of 829 mg U g^−1^ at pH = 7 (Fig. 5d). These results demonstrate the rapid and selective uranium extraction capability of mDSR under near-neutral conditions.

**Figure 5.**
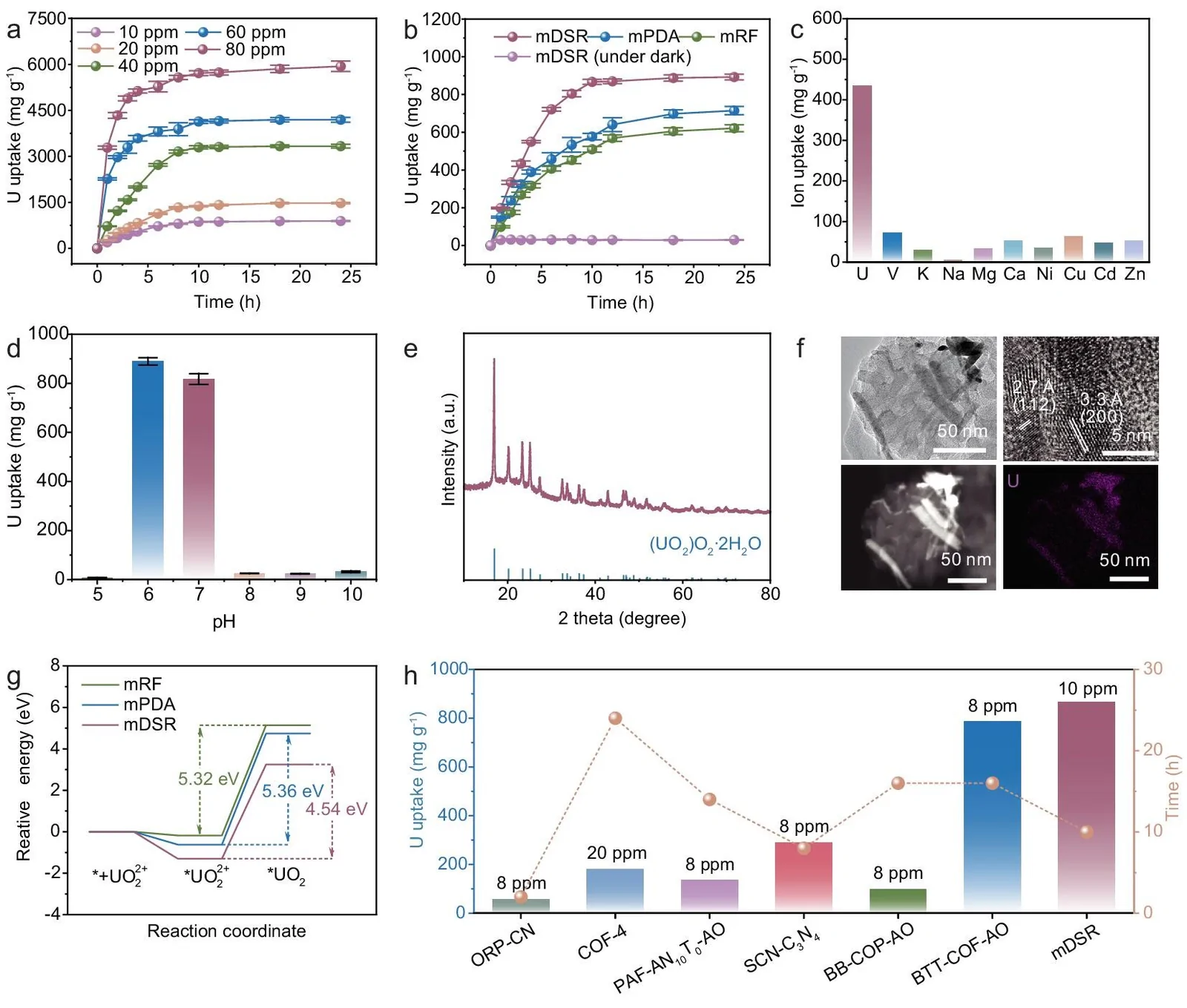
(a) Uranium extraction capacity of mDSR under light irradiation at different initial U(VI) concentrations (10–80 ppm, pH = 6). (b) Uranium extraction capacity of mDSR, mPDA and mRF under light irradiation, and of mDSR in the dark, at an initial U(VI) concentration of 10 ppm (pH = 6). (c) Ion selectivity of mDSR in uranium-spiked seawater (3 ppm). (d) Uranium extraction capacity of mDSR under light irradiation at an initial U(VI) concentration of 10 ppm and at different pH values. (e) The XRD pattern, (f) HRTEM, HAADF-STEM and EDX element mapping images of mDSR after uranium extraction. (g) Calculated free-energy profiles of mDSR, mPDA and mRF before and after uranyl adsorption and after photocatalytic reduction of uranyl. (h) Comparison of the uranium extraction capacity of mDSR with previously reported materials under low uranium concentrations.

The optoelectronic properties of mDSR, mPDA and mRF were subsequently examined to clarify their different photocatalytic behaviors. Analyses of the UV–vis diffuse reflectance spectra using the Kubelka–Munk function yield estimated optical band gaps of 2.47, 1.46 and 2.16 eV for mDSR, mPDA and mRF, respectively (Fig. S20a and b). Combined with the band gap data (Fig. S20b), the energy band diagrams of the materials can be obtained (Fig. S20c), indicating that all materials possess sufficient driving force to reduce U(VI) species to U(IV) species. Electrochemical impedance spectroscopy (Fig. S21) reveals that the charge transfer resistance of mDSR is substantially higher than those of mRF and mPDA. The Mott–Schottky plots (Fig. S22a–c) indicate that materials are all n-type semiconductors in which most of the carriers are electrons [51].

The photophysical properties of the materials were further investigated by steady-state and time-resolved photoluminescence spectroscopy. Among the three materials, mDSR exhibits the highest photoluminescence intensity (Fig. S23a), indicating that mDSR possesses the highest charge carrier concentration. mDSR shows a fluorescence lifetime of 4.82 ns, longer than those of mRF (3.68 ns) and mPDA (0.78 ns) (Fig. S23c and d). These results indicate that the structural features of mDSR promote the generation of photogenerated charge carriers while suppressing electron-hole recombination. Transient photocurrent measurements provide further support for this interpretation. mDSR produces the strongest photocurrent response among the three materials (Fig. S24), indicating the highest effective carrier concentration and charge-separation efficiency. Electron paramagnetic resonance spectroscopy further identifies superoxide radicals (•O_2_^−^) as the predominant reactive oxygen species generated under illumination, with mDSR producing the highest concentration of •O_2_^−^ (Fig. S25). The enhanced generation of superoxide species is favorable for promoting the reduction of uranyl ions [52] and for suppressing microbial adhesion and biofouling in complex seawater environments [18]. Consistent with this result, mDSR exhibits pronounced antibacterial activity in antibacterial assays (Fig. S26).

The samples after extraction were further characterized by HRTEM and X-ray diffraction (XRD) (Fig. 5e and f). The TEM image reveals elongated crystalline domains within the pore channels, while the HRTEM image shows well-resolved lattice fringes with interplanar spacings of approximately 0.27 and 0.33 nm (Fig. 5f). Together with the corresponding XRD pattern (Fig. 5e), the uranium extraction product is identified as (UO_2_)O_2_·2H_2_O, with the measured lattice spacings assigned to its (112) and (200) planes, respectively. HAADF-STEM and EDS elemental mapping images further reveal that abundant, finely dispersed, plate-like uranium-containing species are distributed uniformly throughout the pore channels (Fig. 5f). The analysis of the XPS U 4f region indicates that uranium species in the recovered material exist as a mixture of U(VI) and U(IV) oxidation states (Fig. S27), indicating that the uranyl ions are finally reduced to U(IV) species. Then U(IV) species are probably reoxidized to (UO_2_)O_2_·2H_2_O by air and the resulting phase can then detach from the material surface, potentially regenerating the active sites and enabling a sustainable cycle for uranium extraction from seawater.

Based on these findings, representative molecular fragments of each material were constructed (Fig. S28), and density functional theory calculations were performed to clarify the origin of the superior uranium-extraction performance of mDSR. The free energy diagrams for uranium adsorption and reduction show that the *UO_2_^2+^ intermediate is most strongly stabilized on mDSR, which also exhibits the most favorable energetics for the photocatalytic reduction step (Fig. 5g). The charge-density-difference analyses of *UO_2_^2+^ on mDSR, mPDA and mRF (Fig. S29) further reveal pronounced differences in interfacial charge transfer (Tables S1–S3). The adsorbed uranyl species on mDSR gain ∼0.37 e, substantially exceeding the corresponding values for mPDA (0.22 e) and mRF (0.10 e). These results indicate that, compared with mRF and mPDA, mDSR not only binds uranyl ions more strongly but also promotes their photocatalytic reduction through enhanced charge-transfer capability. Finally, the uranium-extraction performance of mDSR compares favorably with a range of materials specifically developed for seawater uranium extraction (Fig. 5h and Fig. S30 and Table S4) [53–65]. Collectively, these findings confirm that the salen motifs can substantially enhance both metal-ion capture and catalytic activity.

## CONCLUSIONS

In summary, we have developed an ionization-regulated self-assembly strategy for constructing mesoporous salen-based polymers. By controlling precursor ionization, this strategy has been extended to structurally diverse salen ligands, enabling the modular construction of mesoporous polymers with programmable molecular functions, resulting in the ordered mesoporous polymer structures with diverse functionalities. Benefiting from the strong metal-chelating capability of the salen active sites and the intrinsic photocatalytic properties of the conjugated polymer framework, the obtained mDSR exhibits rapid adsorption kinetics and high uranium-extraction performance under illumination, reaching uptake capacities of 867 mg U g^−1^ from a 10 ppm uranyl ion solution and 5715 mg U g^−1^ from an 80 ppm uranyl ion solution within 10 h. This approach provides a general strategy for designing functional mesoporous polymers and expands mesoporous polymers, potential applications in emerging fields.

## Supplementary Material

nwag569_Supplemental_File
