# Supplementary material for: Synthesis of mesoporous salen polymer nanospheres for highly efficient photocatalytic extraction of uranium from seawater
Source: Natl Sci Rev. 2026 Sep 3;13(19):nwag569. doi: 10.1093/nsr/nwag569 (PMC13715444; doi:10.1093/nsr/nwag569)
Supplement: Supplementary file 1 [file nwag569_supplemental_file.pdf]

Supporting information

Synthesis of mesoporous salen polymer nanospheres for highly efficient photocatalytic extraction of uranium from seawater

Yenpin Chu#, Yan Ai#, Yuxuan Li, Jun Wang, Kailin Li, Ziqing Yin, Xiaomin Li， Tongtao Li, Dongyuan Zhao, Wei Li*

Department of Chemistry, Laboratory of Advanced Materials, Shanghai Key Laboratory of Molecular Catalysis and Innovative Materials, and State Key Laboratory of Porous Materials for Separation and Conversion, Fudan University, Shanghai 200433, China

***Corresponding author.** E-mail: weilichem@fudan.edu.cn

# Equally contributed to this work.

Experimental Section.

Chemicals and Materials.

The triblock copolymer Pluronic F127 ($EO_{106}PO_{70}EO_{106}$), F108 ($EO_{132}PO_{50}EO_{132}$), P123 ($EO_{20}PO_{70}EO_{20}$) and uranyl nitrate hexahydrate were purchased from Sigma-Aldrich. o-phenylene-diamine (OPD), (1R,2R)-(-)-1,2-diaminocyclohexane, 2,6-diaminopyridine (DAP), tetramethylammonium hydroxide pentahydrate (TMAOH), resorcinol, dopamine hydrochloride (DA) and Nafion were purchased from Aladdin Chemical Corp. 2,4-dihydroxybenzaldehyde was purchased from Shanghai Adamas Reagent Corp. Ammonium chloride (NH_4Cl), ammonium sulfate ($(NH_4)_2SO_4$), hexamethylene-tetramine (HMTA), sodium hydroxide (NaOH), ethylenediamine, diethylenetriamine (DETA), 1,3,5-trimethylbenzene (TMB), anhydrous ethanol, anhydrous methanol, sodium chloride (NaCl), acetic acid, hydrochloric acid (HCl, 37 wt. %), nitric acid (HNO_3, 68 wt. %), formaldehyde aqueous solution, ammonium hydroxide ($NH_3 \cdot H_2O$, 28-30 wt. %) and triethylamine were purchased from Shanghai Chemical Corp. All the reagents were directly used without further purification.

Synthesis of the Salen/Salen-type ligands

(1) Synthesis of N,N'-bis(2,4-dihydroxybenzylidene)ethylenediamine (denoted as Salen).

Anhydrous ethylenediamine (2.0 mL, 30 mmol) was added to a 250 mL three-necked round-bottom flask containing 20 mL of anhydrous ethanol. The mixture was heated to reflux (78-80 °C) in an oil bath. Separately, 2,4-dihydroxybenzaldehyde (60 mmol) was dissolved in 30 mL of anhydrous ethanol and added dropwise to the refluxing solution at a rate of 2-3 drops per second. The mixture was then heated under reflux for an additional 12 h. After completion, the reaction was cooled in an ice-water bath for 1 h. The solid was collected by vacuum filtration, washed several times with a small amount of anhydrous ethanol, and dried in an oven at 60 °C for 2 h to afford the Salen. (^{1}H NMR (DMSO-d_6, 500 MHz, ppm); δ: 13.55 (br, 2H), 9.67 (br, 2H), 8.36 (s, 2H), 7.16 (d, J = 8.4 Hz, 2H), 6.26 (dd, J = 8.4 and 2.4 Hz), 6.17 (d, J = 2.4 Hz, 2H), 3.78 (s))，yield~90 %

(2) Synthesis of N,N'-bis(2,4-hydroxybenzylidene)-1,2-phenylenediamine (denoted as OPD-Salen).

The synthesis was conducted following a procedure analogous to that described for Salen, with the exception that ethylenediamine was replaced with OPD and several

drops of acetic acid were added dropwise during the reflux process to catalyze the reaction. (^{1}H NMR (DMSO-d_6, 500 MHz, ppm δ: 13.39 (s, 2H), 10.27 (s, 2H), 8.75 (s, 2H), 7.43 (d, J = 8.5 Hz, 2H), 7.35 (dt, J = 7.1, 3.6 Hz, 2H), 7.35–7.30 (m, 2H), 6.40 (dd, J = 8.5, 2.3 Hz, 2H), 6.29 (d, J = 2.2 Hz, 2H)), yield~70 %

(3) Synthesis of (1R,2R)-N,N'-bis(2,4-hydroxybenzylidene)-1,2-diaminocyclohexane (denoted as Chiral-Salen).

The synthesis was conducted following a procedure analogous to that described for Salen, with the exception that ethylenediamine was replaced with (1R,2R)-(-)-1,2-diaminocyclohexane. (^{1}H NMR (DMF-d_7, 500 MHz, ppm); δ: 13.54 (br, 2H), 8.35 (s, 2H), 7.15 (d, J = 8.4 Hz, 2H), 6.32 (dd, J = 8.4 and 2.4 Hz), 6.25 (d, J = 2.4 Hz, 2H), 3.41 (4H, s), 1.8-1.9 (4H, m), 1.48-1.63 (4H, m)), yield~91 %

(4) Synthesis of a Salen-type ligand bridged by 2,6-diaminopyridine (denoted as DAP-Salen).

2,6-Diaminopyridine (0.12 mol) was added to a 500 mL flask and dissolved in 260 mL methanol (denoted as solution A). Separately, 2,4-dihydroxybenzaldehyde (0.24 mol) was added to another flask and dissolved in 100 mL of methanol (denoted as solution B). Solution B was then added dropwise to solution A at a rate of 2-3 drops per second using a dropping funnel. Upon completion of the addition, the dropping funnel was rinsed with a small amount of methanol. Approximately 0.3 mL of acetic acid was added to serve as a catalyst for the reaction. An orange solid began to precipitate after about 2-3 h of reaction. The mixture was allowed to react under continously stirring for an additional 36 h. After the reaction was complete, triethylamine (0.75 mL) was added to neutralize the acetic acid. The flask was then cooled in an ice-water bath for 1 h, and the resulting precipitate was collected by vacuum filtration. The solid was thoroughly washed by anhydrous ethanol for three to four times. Finally, the product was dried at a low temperature of 40 °C to remove residual ethanol, affording the DAP-Salen, yield~93%. No obvious NMR signals were observed in the ^{1}H NMR spectrum (DMSO-d_6, 500 MHz, ppm), which was consistent with the work of Milosevic et al.[1]. MS (ESI): m/z 350.1147 $[M+H]^+$. The calculated molecular formula based on the nitrogen rule was $C_{19}H_{15}N_3O_4$, which matched the expected molecular formula.

(5) Synthesis of a Salen-type ligand bridged by diethylenetriamine (denoted as DETA-Salen).

Diethylenetriamine (30 mmol) was placed in a mortar. 2,4-Dihydroxybenzaldehyde (60 mmol) was then added to the mortar in several small portions with

continuous, rapid grinding to ensure thorough contact and reaction between the two components. A viscous orange solid formed immediately upon addition. This solid was repeatedly scraped down from the mortar walls with a spatula and grinding was continued. The viscous orange solid gradually transformed into a loose, bright orange-yellow powder. Once no viscous material was visibly observed, grinding was continued for an additional 30 min to ensure complete reaction. The resulting powder was thoroughly washed by anhydrous ethanol for three to four times. Finally, the product was dried in an oven at 60 °C, affording the DETA-Salen. (^{1}H NMR (DMSO-d_6, 500 MHz, ppm); δ: 8.26 (s, 2H), 7.10 (d, J = 8.4 Hz, 2H), 6.20 (dd, J = 8.4, 1.7 Hz, 2H), 6.12 (d, J = 2.1 Hz, 2H), 3.54 (t, J = 5.9 Hz, 4H), 2.79 (t, J = 6.1 Hz, 4H)), yield~97 %.

Synthesis of mesoporous DAP-Salen-formaldehyde resin spheres (denoted as DSR).

(1) Synthesis of DAP-Salen-based mesoporous polymer spheres with highly ordered vertically aligned mesopores (denoted as mDSR).

In a typical experimental procedure, 1 g of Pluronic F127 and 2.5 g of NH_4Cl and 0.5 mL of TMB were successively dissolved in a mixture consist of 20 mL of ethanol and 40 mL of deionized water. And the solution was vigorously stirred at 900 rpm for 15 min. Then, 3 g of TMAOH and 0.6 g of HMTA was introduced, and the pH was adjusted to approximately 9.3 by adding 0.6 mL of $NH_3·H_2O$. The stirring was continued at 900 rpm for approximately 8 h to allow thorough formation of the microemulsion. 1 g of DAP-Salen was then added, and the mixture was sonicated for 5 min with simultaneous shaking to ensure dispersion. Stirring was resumed at 900 rpm for an additional 3.5 h to ensure suitable ionization of the DAP-Salen. Finally, the reaction mixture was stirred at 80 °C. After 24 h, the mixture was cooled to room temperature and was collected by centrifugation at 13,000 rpm for 10 min. The collected solid was washed several times with a water/ethanol mixture and dried in an oven at 60 °C, affording mDSR, yield~91 % The amount of TMB can be changed from 0.5 mL to 3 mL.

(2) Mesoporous polymer spheres synthesized with different $NH_4Cl/(NH_4)_2SO_4$ ratios.

The synthesis procedure was identical to that described for mDSR, except that different $NH_4Cl/(NH_4)_2SO_4$ ratios were employed, with the total molar amount of ammonium ions kept equal to that in the standard 2.5 g NH_4Cl synthesis.

For the F108 system, the procedure remained the same, except that F127 was replaced with an equimolar amount of F108.

Synthesis of mesoporous Salen-formaldehyde resin spheres (denoted as mSR).

In a typical experimental procedure, 1 g of Pluronic F127 and 2.5 g of NH_4Cl and 1.5 mL of TMB were successively dissolved in a mixture consist of 20 mL of ethanol and 40 mL of deionized water. And the solution was vigorously stirred at 900 rpm for 15 min. Then, 0.4 g of NaOH and 0.3 g of HMTA was introduced. Stirring was continued at 900 rpm for approximately 8 h to allow thorough formation of the microemulsion. 0.33 g of Salen was then added, and the mixture was sonicated for 5 min with simultaneous shaking to ensure dispersion. Stirring was resumed at 900 rpm for an additional 6 h to ensure suitable ionization of the Salen. Finally, the reaction mixture was stirred at 80 ^{O}C. After 24 h, the mixture was cooled to room temperature and was collected by centrifugation at 13,000 rpm for 10 min. The collected solid was washed several times with a water/ethanol mixture and dried in an oven at 60 °C, affording mSR.

The synthesis procedures for other Salen/Salen-type-based mesoporous polymers were identical to that described for the previous material, except that the precursor was replaced with an equimolar amount of the corresponding ligand, without any further optimization.

Synthesis of mesoporous resorcinol-formaldehyde resin spheres (denoted as mRF).

0.9 g of Pluronic F127 and 0.36 g of resorcinol and 1.5 mL of TMB were successively dissolved in a mixture consist of 30 mL of ethanol and 30 mL of deionized water and 1.2 mL of $NH_3 \cdot H_2O$ by stirring at 600 rpm for 30 min to form a micelles system. Then, 0.45 mL of formaldehyde aqueous solution was introduced to initiate polymerization, and the reaction was allowed to proceed with continued stirring at 700 rpm. After 8 h, the reaction was quenched. The product was collected by centrifugation at 10,000 rpm for 6 min, washed several times with a water/ethanol mixture, and dried in an oven at 60 °C, affording the product, denoted as mRF.

Synthesis of mesoporous polydopamine spheres (denoted as mPDA).

The mPDA was prepared according to the previously reported method[2]. 0.6 g of F127, 0.3 g of DA and 2 mL of TMB were successively dissolved in a mixture consist of 30 mL of ethanol and 30 mL of deionized water by stirring at 600 rpm for 30 min to

form a micelles system. Then, 0.5 mL of $NH_3·H_2O$ was added to induce the polymerization of DA precursor. The reaction was allowed to proceed for 24 hThe product was collected by centrifugation at 12,000 rpm for 8 min, washed several times with a water/ethanol mixture, and dried in an oven at 60 °C, affording the product, denoted as mPDA.

Materials Characterization.

The nitrogen sorption isotherms were measured using the Micromeritics ASAP 2460. Prior to measurements, the materials were degassed in a vacuum at 140 °C for 12 h. Scanning electron microscopy images were captured using a Zeiss Gemini 560 microscope with an accelerated voltage of 1 kV. Transmission electron microscopy images were acquired using a Thermo Fisher Talos F200S G2 microscope and a JEOL JEM-F200. Infrared spectra were recorded using a Nicolet iS10 from ThermoFisher. X-ray photoelectron spectroscopy (XPS) was performed using a PHI Genesis 500. Small-angle X-ray scattering (SAXS) measurements was performed using a Xenocs Xeuss2.0, using Cu-Kα radiation (50 kV, 0.6 mA). Solid-state ^{13}C cross-polarization magic-angle spinning (^{13}C CP-MAS NMR) spectra was performed using a Bruker AVANCE NEO 600WB, and ^{13}C NMR and ^{1}H NMR spectra was performed using a Bruker 600 MHz AVANCE NEO. Mass spectra were recorded using a ACQUITY UPC2-Xevo G2-XS TOF. Wide-angle X-ray diffraction (XRD) patterns were obtained using a Bruker D8 Xray diffractometer (Germany) with Cu-Kα radiation (40 kV, 40 mA). Metal ions concentrations were determined using an PE-8000 inductively coupled plasma mass spectrometry (ICP-MS). Elemental analyses were obtained using a Elementar UNICUBE. Diffuse reflectance spectra were recorded using a Lambda 1050+. Photoluminescence emission spectra and time-resolved fluorescence spectra were obtained using a FLS1000 fluorescence spectrometer. Photoluminescence emission spectra were measured from 400 to 750 nm under 337 nm excitation by an Xe lamp. Electron paramagnetic resonance (EPR) spectra were recorded on a Bruker E500 spectrometer.

Photocatalytic reduction of uranium.

A 300 W Xe lamp (PLS-SXE 300+, 320 nm $\leq \lambda \leq$ 780 nm, light intensity 1 kW m^{-2}) was used as the source of simulated sunlight. Reactions were carried out in a 1000 mL photoreactor cooled by circulating water (25±2 ºC). The pH value of the solutions was adjusted between 5 and 10 with aqueous HNO_3 or NaOH solution. 5 mg of catalyst

was suspended in 500 mL of a solution containing U(VI) ion (10~80 ppm, from uranium nitrate). Solution was sampled at regular intervals from the stirring mixture. The concentration of the solution was determined using a UV spectrophotometer and Beer–Lambert law, according to equation (1):

$$A = \log\frac{1}{T} = Kbc \qquad (1)$$

where A is the absorbance of the solution, T is the transmittance of the solution, K is the molar absorption coefficient, b is the thickness of the absorbing layer, and c is the concentration of the substrate. Arsenazo was used as the imaging agent, 0.5 mL of the solution was added into a mixture of 1 mL of azoarsenic (500 mg L^{-1}) and 0.25 mL of HCl (0.1 mol L^{-1}), followed by dilution with 3.25 mL of water.

The adsorption uptake of the sample was calculated using equation (2):

$$q_e = \frac{(C_0 - C_e)\times V}{m} \qquad (2)$$

where C_0 and C_e (mg L^{-1}) are the uranium concentrations in the initial and equilibrium states, respectively; V (L) is the solution volume; m (g) is the weight of the COF powder; and q_e (mg g^{-1}) is the adsorption uptake.

The adsorption uptake curves of the catalyst at different concentrations were fit using Langmuir and Freundlich models.

Langmuir model:

$$\frac{C_e}{q_e} = \frac{1}{k_L} + \frac{C_e}{q_{max}} \qquad (3)$$

Freundlich model：

$$\ln q_e = \ln k_F + \frac{1}{n} C_e \qquad (4)$$

where C_e (mg L^{-1}) is the equilibrium concentration of the material, q_e (mg g^{-1}) is the equilibrium adsorption uptake, q_{max} (mg g^{-1}) is the maximum adsorption uptake, and k_L and k_f (mg L^{-1}) are the Langmuir and Freundlich binding constants, respectively.

Selectivity studies.

5 mg of catalyst was added to 500 mL of a solution containing 3 ppm each of 3 ppm UO_2^{2+}, Na^+, K^+, Mg^{2+}, Ca^{2+}, Ni^{2+}, Cu^{2+}, Cd^{2+}, Zn^{2+} and V^{5+}. After stirring for 24 h, the concentrations of metal ions were determined using an inductively coupled plasma atomic emission spectrometer.

The band gap energy

Diffuse reflectance spectra were used to determine the band gap energy of the catalysis. Diffuse reflectance spectra were plotted with reflectance or absorbance as the vertical axis versus wavelength as the horizontal axis. To determine the band gap, equation 5 was used to transform wavelength into energy as a new horizontal axis. Kubelka-Munk equations (equation 6 and 7) transformed the absorbance and reflectance into a new vertical axis. The band gap energy was determined by the tangent of the plot with the horizontal axis[3].

$$E = hv = \frac{hc}{\lambda} \qquad (5)$$

$$(F(R)hv)^r = B(hv - E_g) \qquad (6)$$

$$F(R) = \alpha \qquad (7)$$

where E is the photon energy (eV), h is Planck's constant (4.132×10^{-15} eV·s), ν is the frequency (s^{-1}), c is the photon velocity (nm), λ is the wavelength (nm), α is the absorption coefficient from the absorption mode, B is a constant, E_g is the band gap (eV), r = 2。

Photoelectrochemical characterization

Photoelectrochemical experiments were performed according to the reported methods[4]. All tests were carried out on an electrochemical workstation (CHI660E) using a conventional three-electrode system with platinum as the counter electrode and Hg/ Hg_2Cl_2 as the reference electrode and the working electrode. An aqueous 0.2 M Na_2SO_4 solution was used as an electrolyte. For the preparation of the working electrode, indium tin oxide (ITO) glass was cut into a 0.5 cm × 2 cm. 5 mg of catalysts, 50 μL of Nafion and 950 μL of ethanol were mixed and treated with ultrasound for 30 min to obtain a slurry. Then, 100 μL of slurry was spread onto the ITO glass.

For photocurrent measurements, visible light was generated using a 300 W xenon lamp (PLS-SXE 300+, 320 nm ≤ λ ≤ 780 nm), and the photocurrent response of the photocatalyst was measured without a bias voltage over the course of an "on-off' cycling experiment, where the light was turned on for 60 s and then off for 60 s for 7 cycles.

For Mott-Schottky experiments, the perturbation signal was 5 mV and data were collected at frequencies of 500, 750, and 1000 Hz. The applied potentials vs. SCE were converted to NHE potentials using equation 8.

$$E_{NHE} = E_{SCE} + E_{SCE}^{\theta}(E_{SCE}^{\theta} = 0.2415\,V) \qquad (8)$$

For electrochemical impedance spectroscopy measurements, the applied sine

wave potential amplitude is 5 mV with AC amplitude in the frequency range of 0.01 to 10^6 Hz.

Computational methods

The interactions between uranyl ions/uranium dioxide and the polymer fragments were calculated using density functional theory (DFT) as implemented in the Vienna Ab initio Simulation Package (VASP) [5-7].The electron-ion interactions were described using a plane-wave basis set with a kinetic energy cutoff of 520 eV. The exchange–correlation terms of the correlation functions were exchanged based on the generalized gradient approximation of Perdew-Burke-Ernzerhof (PBE). To balance accuracy and computational efficiency, a vacuum layer of at least 10 Å was introduced. The convergence criterion of self-consistent iteration and the ion relaxation were set at 1×10^{-6} eV and 0.03 eV $Å^{-1}$ to ensure the geometric configuration was sufficiently relaxed. A gamma k-point mesh of 1×1×1 for the Brillioun zone sampling for structural optimization. Furthermore, solvation effects were taken into account by setting the dielectric constant to 78.5, while keeping all other parameters unchanged[8, 9].

The Gibbs free energy change for each elementary step was calculated using the following equation:

$$\Delta G = \Delta E + \Delta E_{ZPE} - T\Delta S$$

Where T was temperature and was taken as 298 K, E was the single-point energy， E_{ZPE} was the zero-point vibrational energy, and S was the entropy.

To further quantify the electron transfer process and catalytic activity between uranyl ions and the three supports, we performed analyses using charge density differences and Bader charges. The isosurface level for the charge density difference was set to 0.005 $|e^-|$, and the number of sampling points for Bader charge analysis was 1000. The Bader charge transfer was calculated according to the following equation:

$$\Delta|e^-| = |e^-|_{bader} - |e^-|_{origin}$$

Figures.

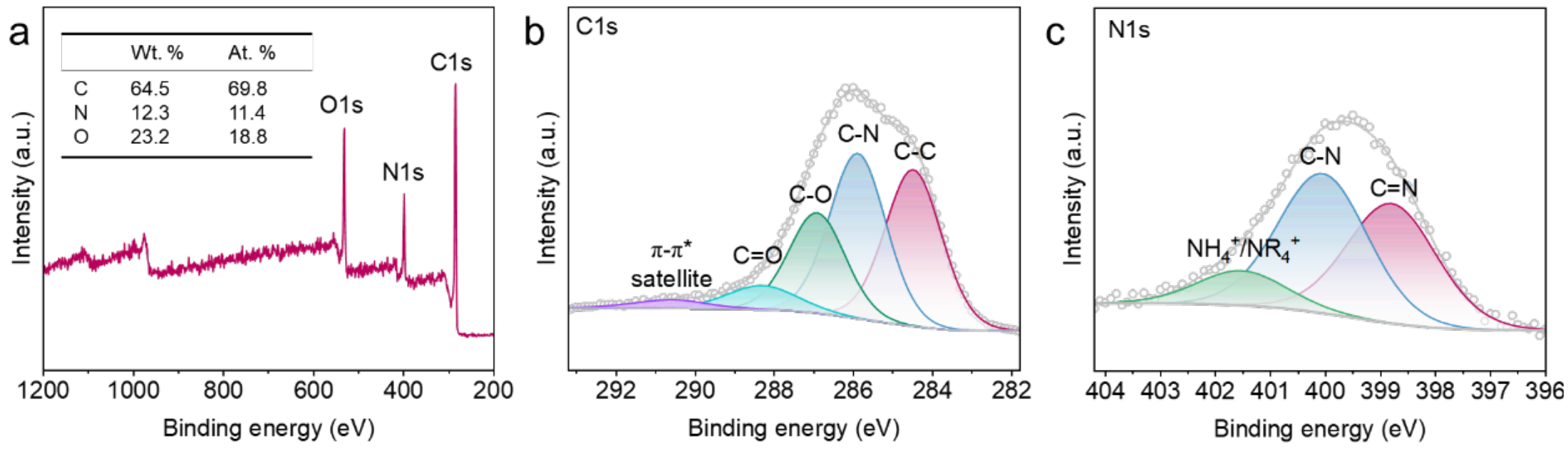

Figure S1. (a) XPS survey spectrum and semi-quantitative analysis results, (b) High-resolution C 1s XPS spectrum and(c) High-resolution N 1s XPS spectrum of mDSR.

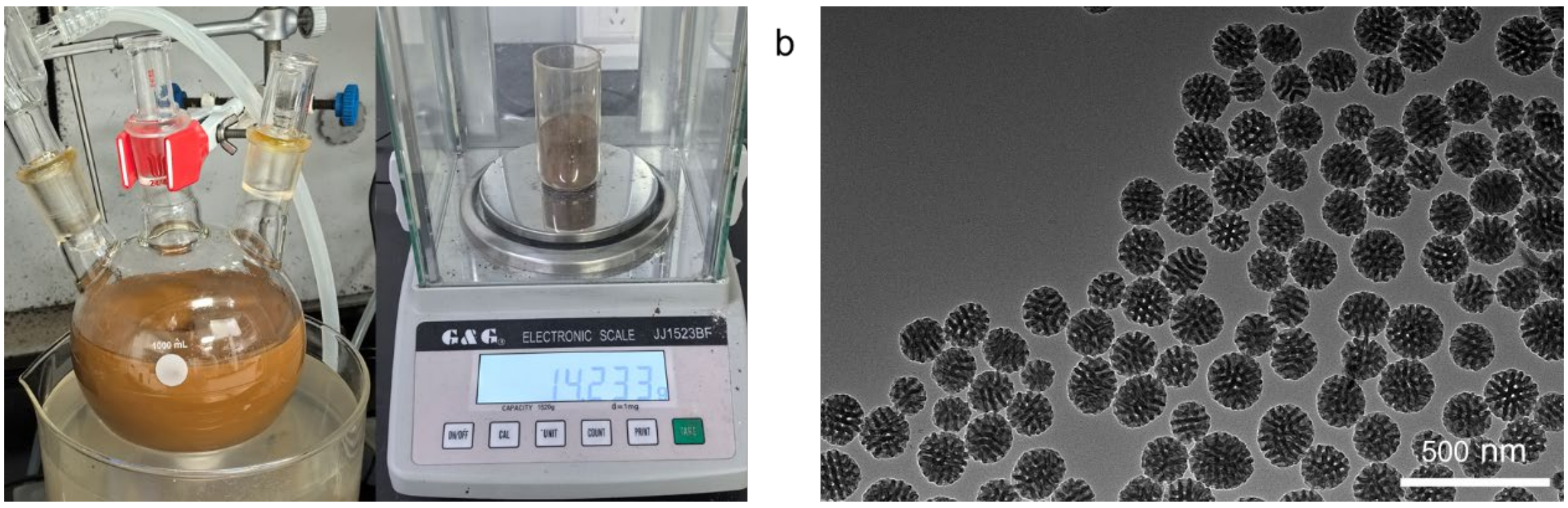

Figure S2. (a) The scale-up synthesis of the mDSR, (b) The TEM image of the samples obtained from scale-up synthesis.

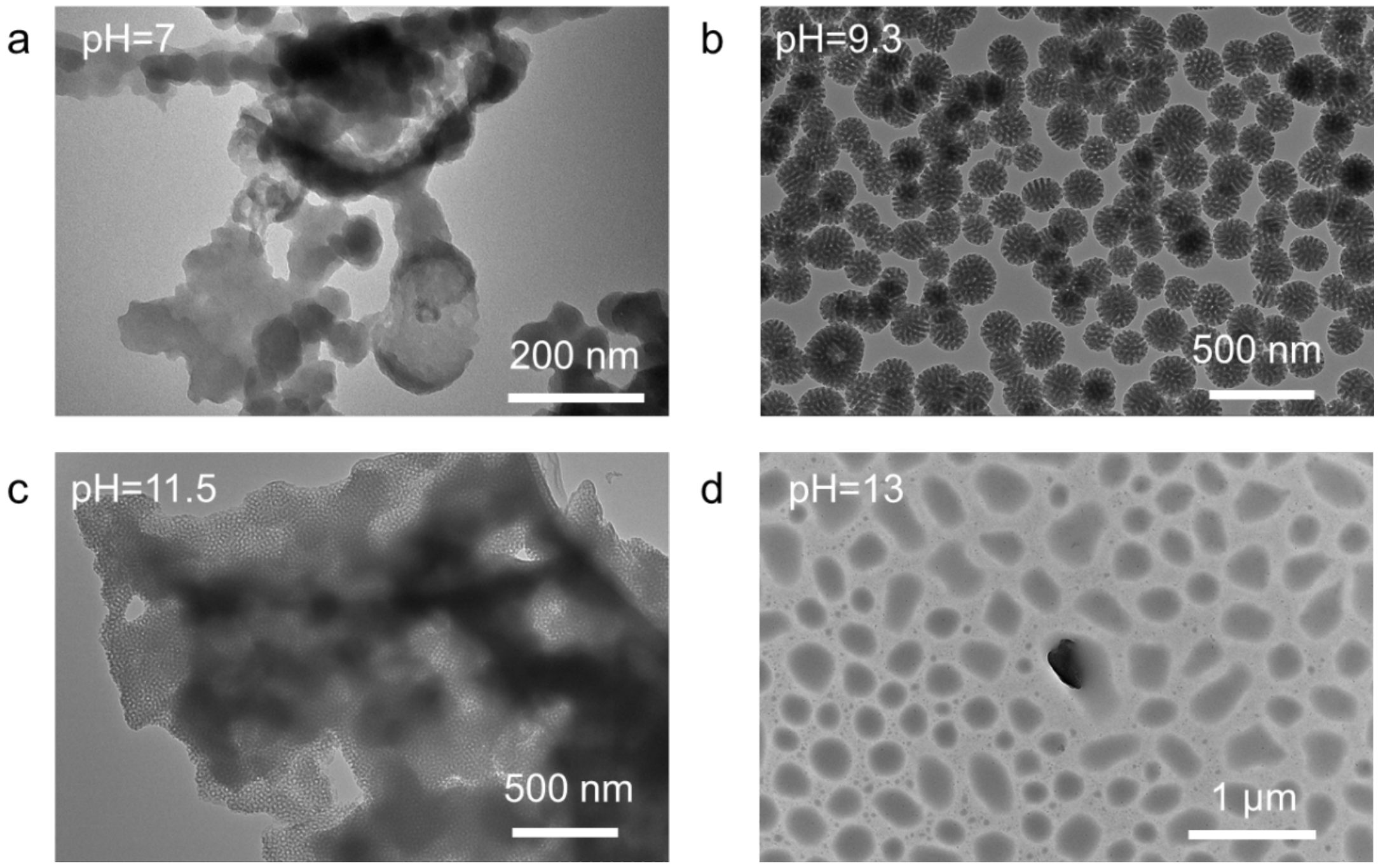

Figure S3. Morphological changes of mDSR synthesized at different pH (a) pH=7, (b) pH=9.3, (c) pH=11.5, (d) pH=13.

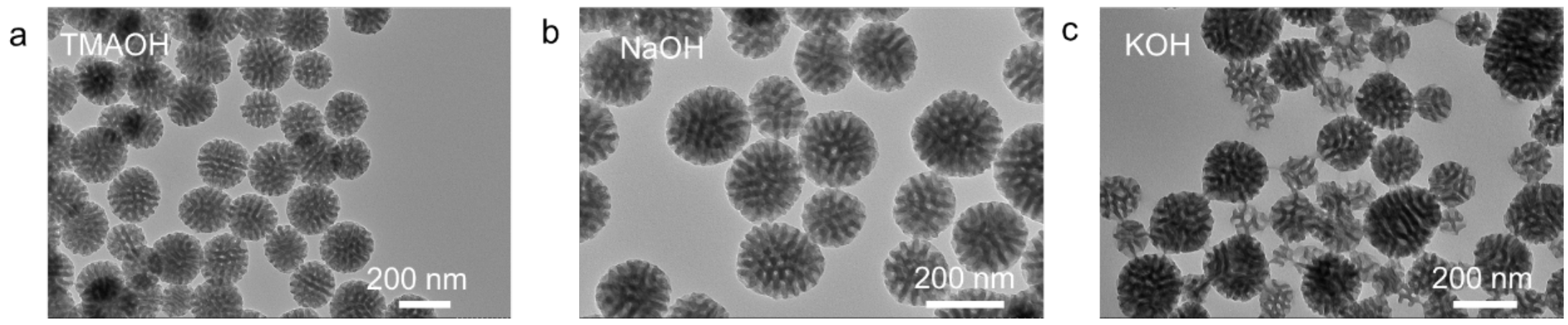

Figure S4. TEM images of mDSR synthesized by different base (a) TMAOH, (b) NaOH, (c) KOH (due to the low solubility of KCl, pH =8.8, which cannot reach 9.3).

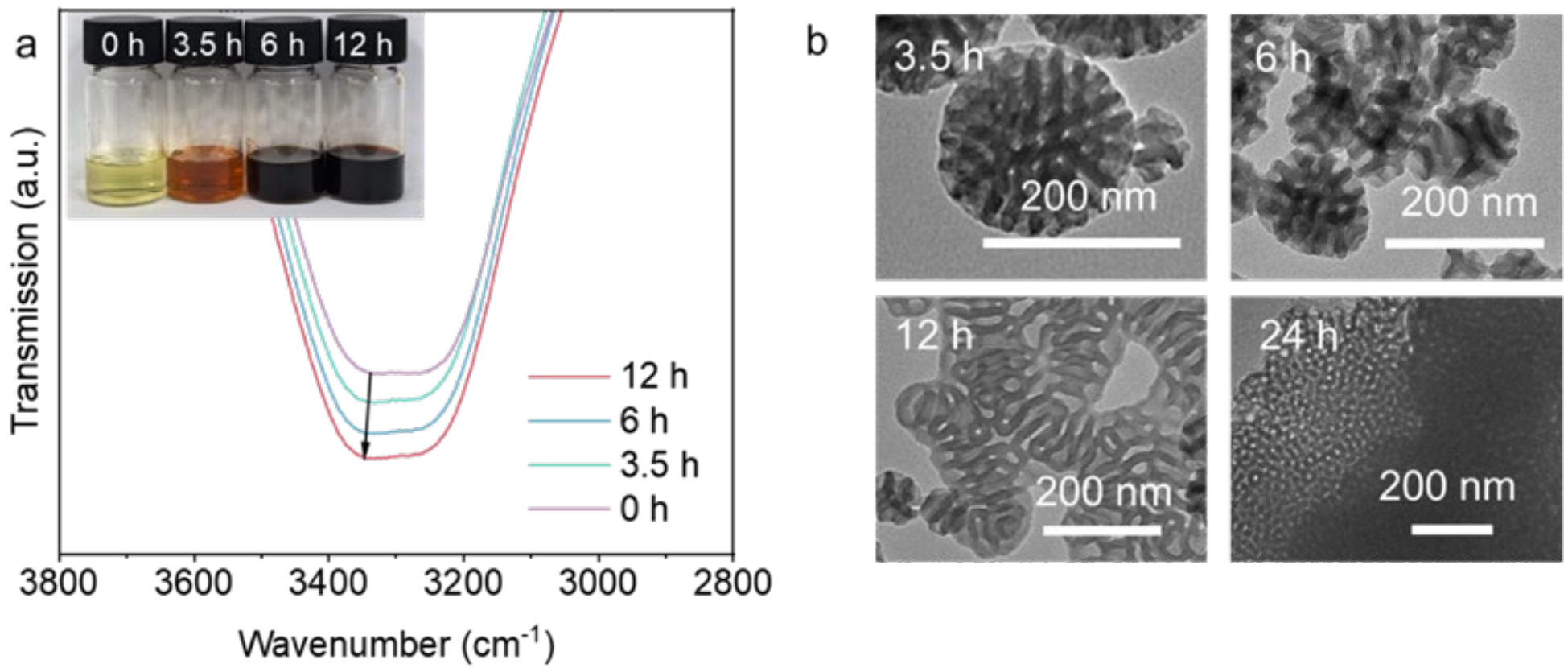

Figure S5. (a) FT-IR spectra of the filtrate obtained after ionizing DAP-Salen in systems with different pre-stirring time (inset: optical photographs of the filtrate obtained after pre-stirring DAP-Salen in systems with different time). (b) Morphological changes of mDSR synthesized from DAP-Salen after being ionized in systems with different pre-stirring time.

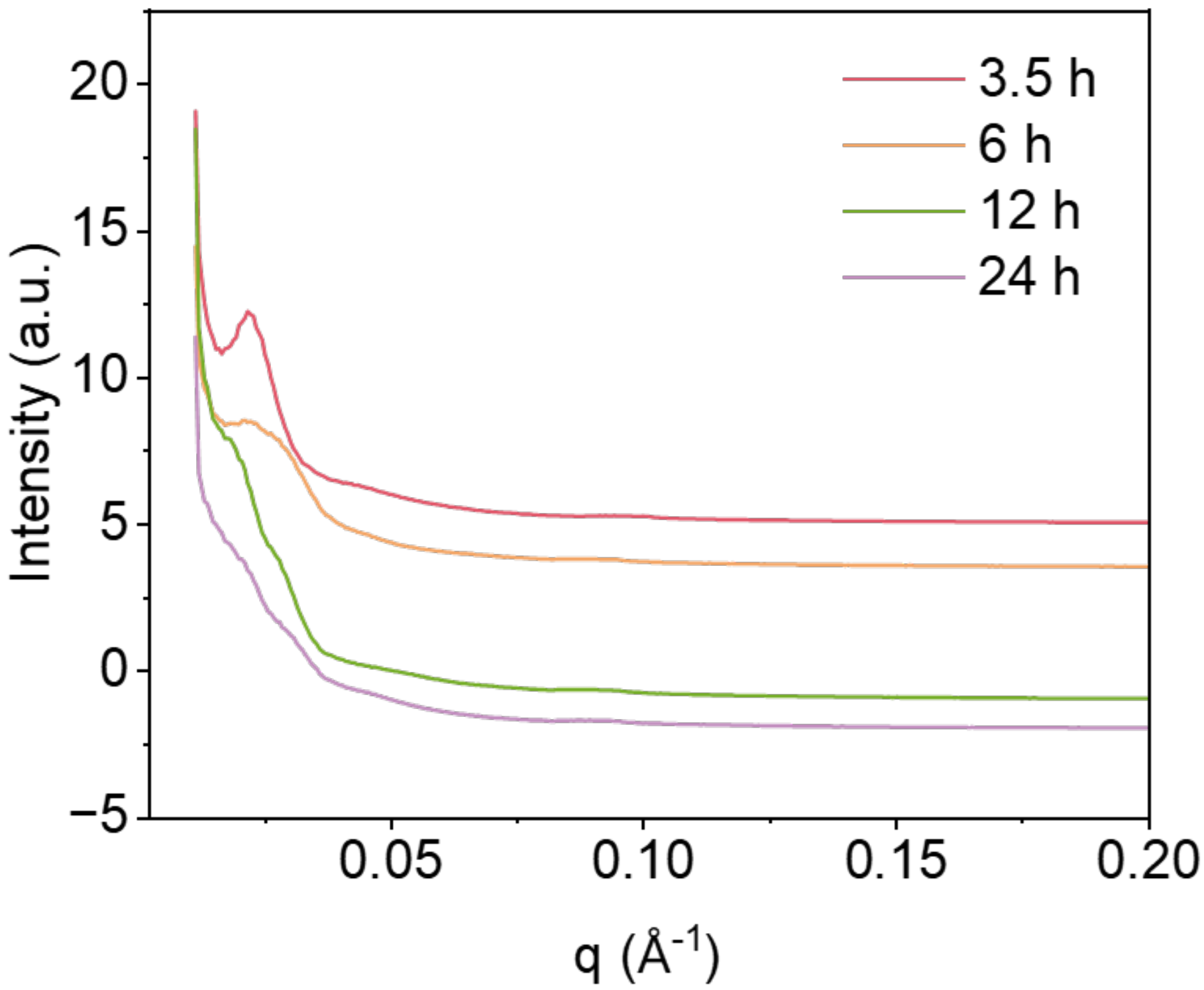

Figure S6. SAXS patterns of mDSR obtained with different pre-stirring time.

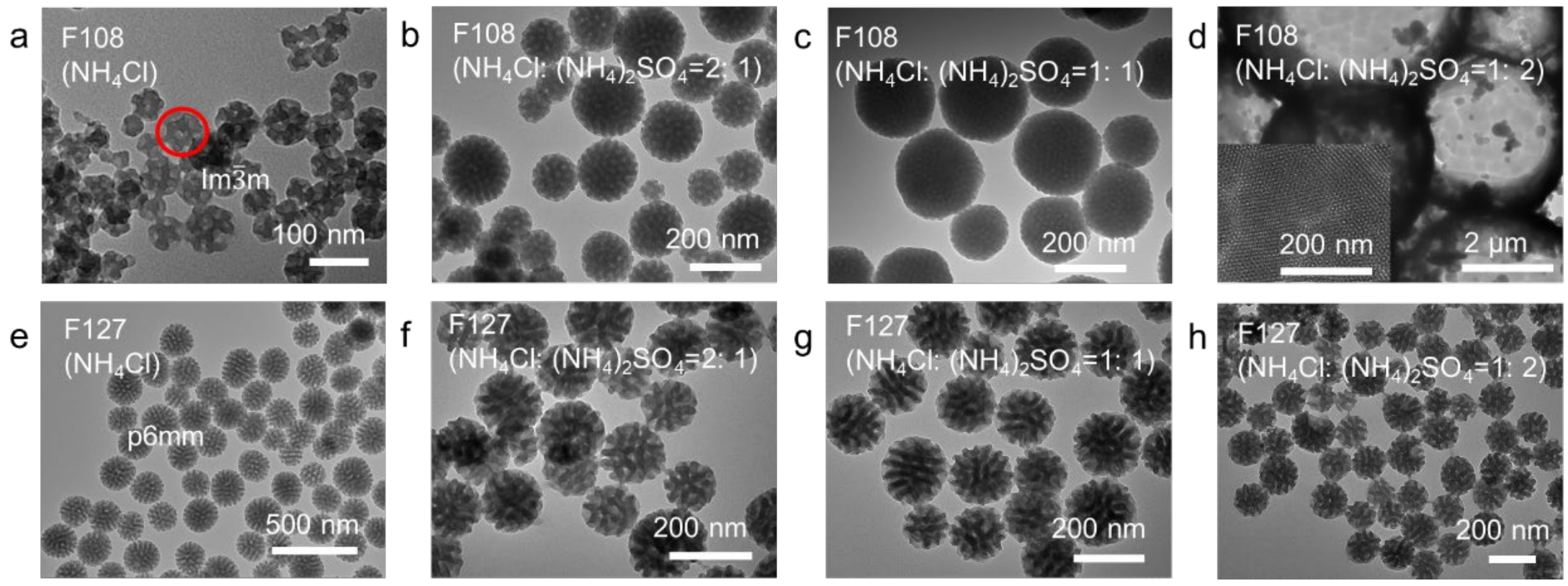

Figure S7. Morphological changes of DSR synthesized with different NH_4Cl/ $(NH_4)_2SO_4$ ratios and different surfactants (a-d) F108, (e-h) F127.

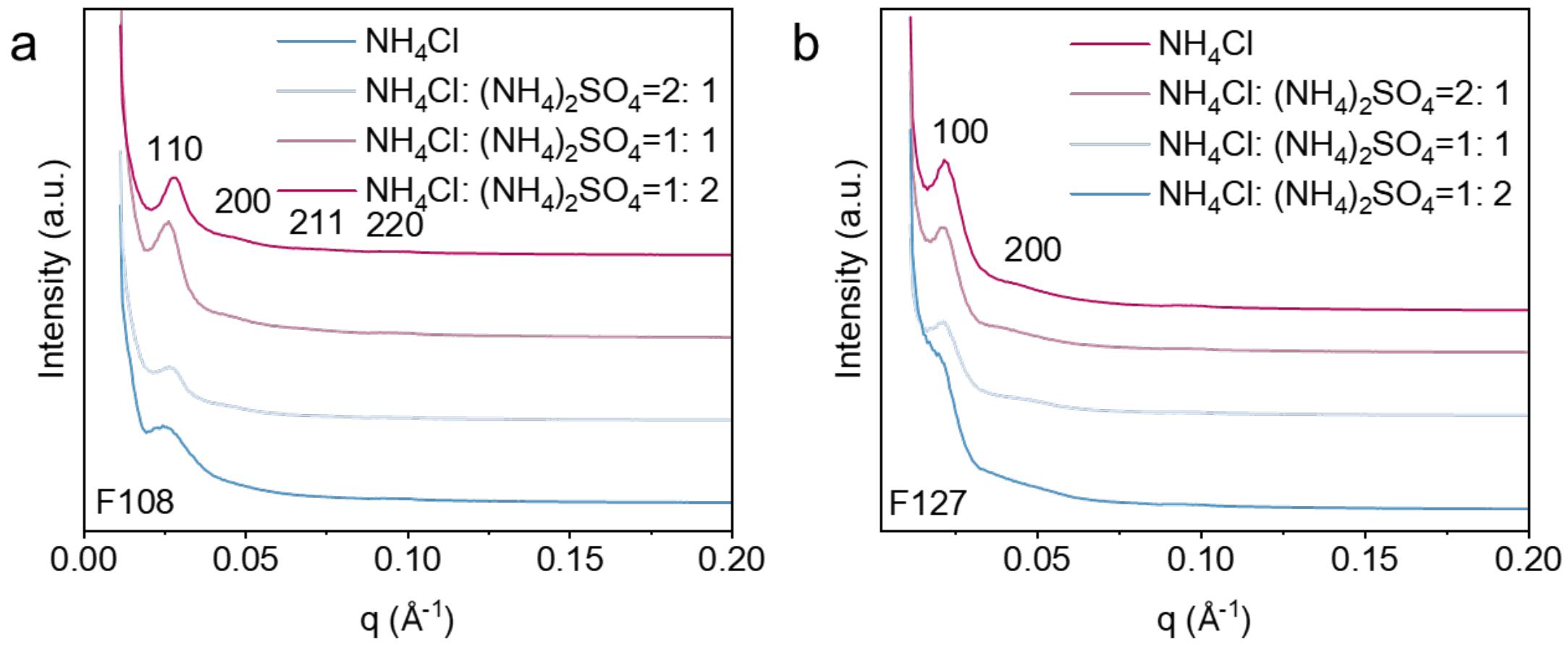

Figure S8. SAXS patterns of mDSR obtained with different $NH_4Cl/(NH_4)_2SO_4$ ratios and surfactants (a) F108, (b) F127.

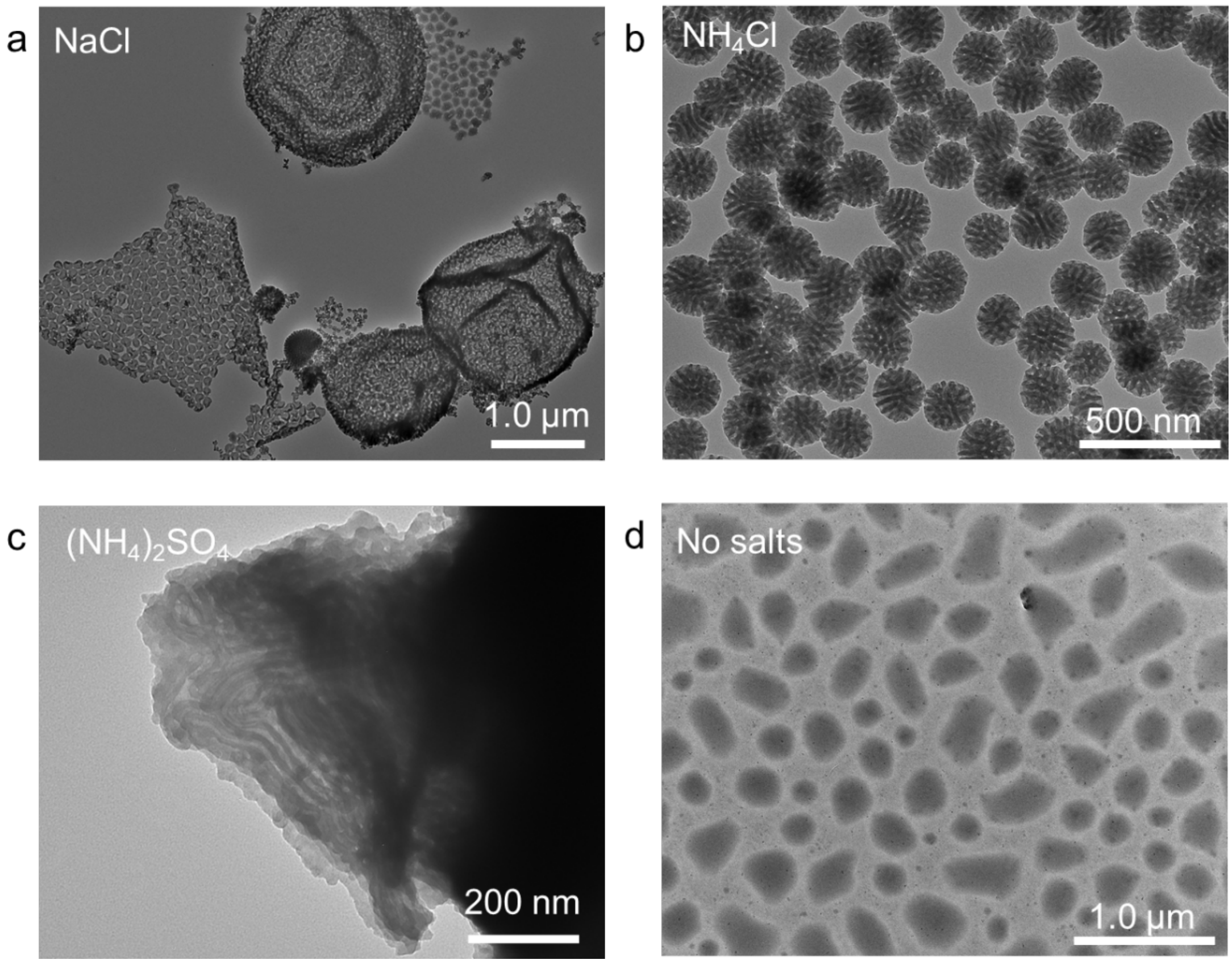

Figure S9. Morphological changes of DSR synthesized with different salts.

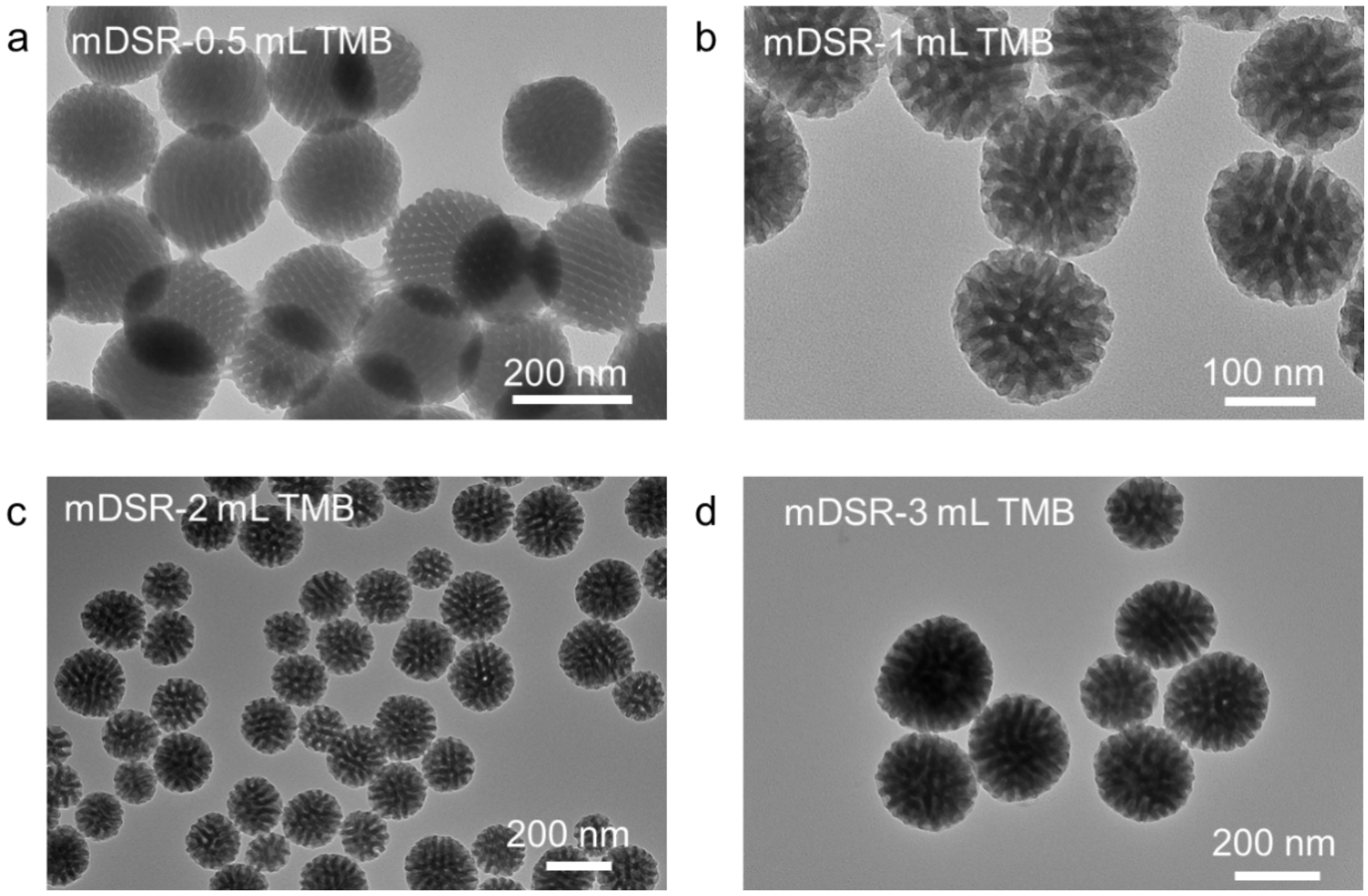

Figure S10. TEM images of mDSR prepared by the ionization modulation approach with different amounts of TMB (a) 0.5 mL, (b) 1.0 mL, (c) 2 mL and (d) 3.0 mL

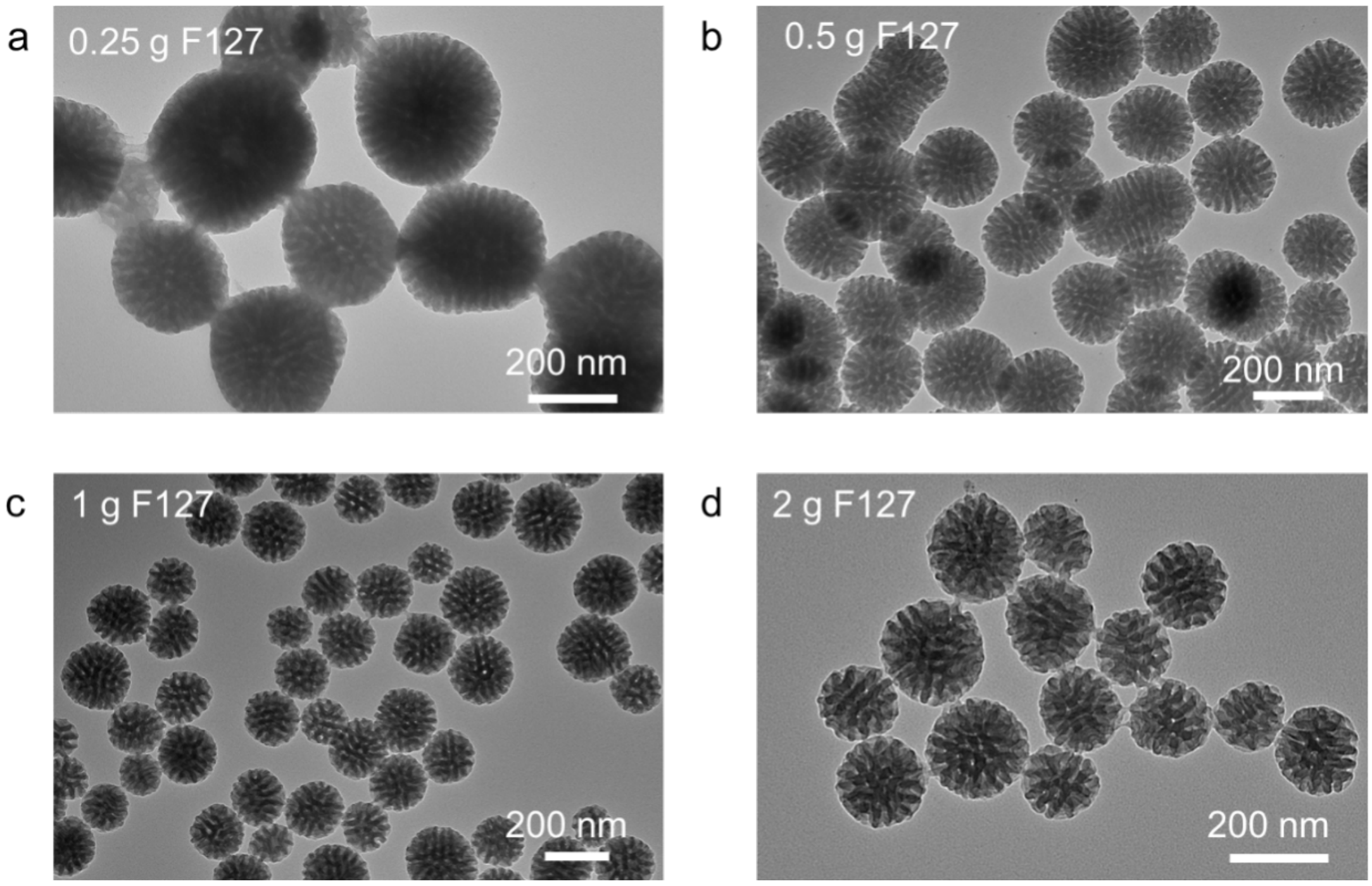

Figure S11. TEM images of mDSR obtained with different amounts of surfactants.

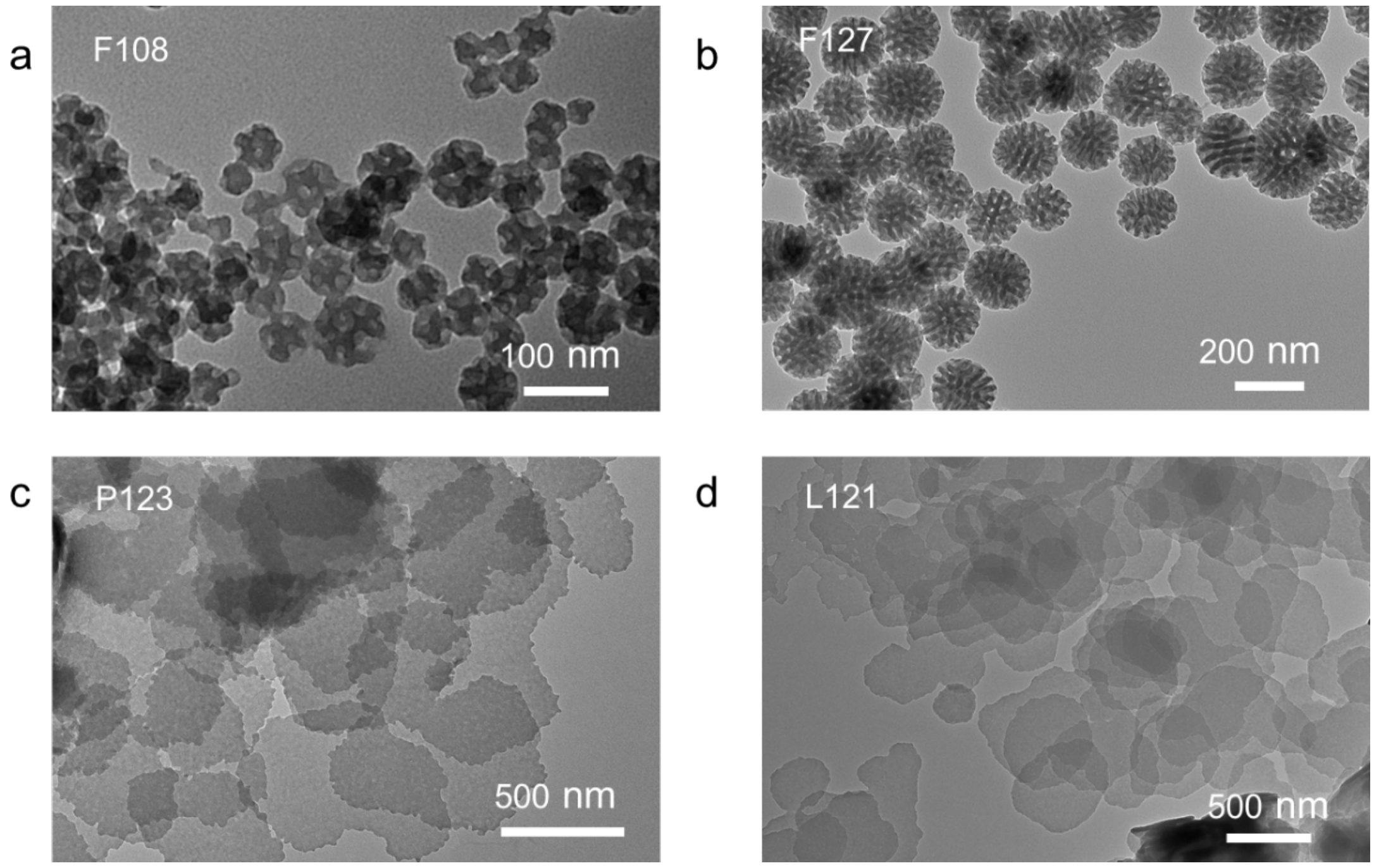

Figure S12. TEM images of mDSR obtained with different surfactants.

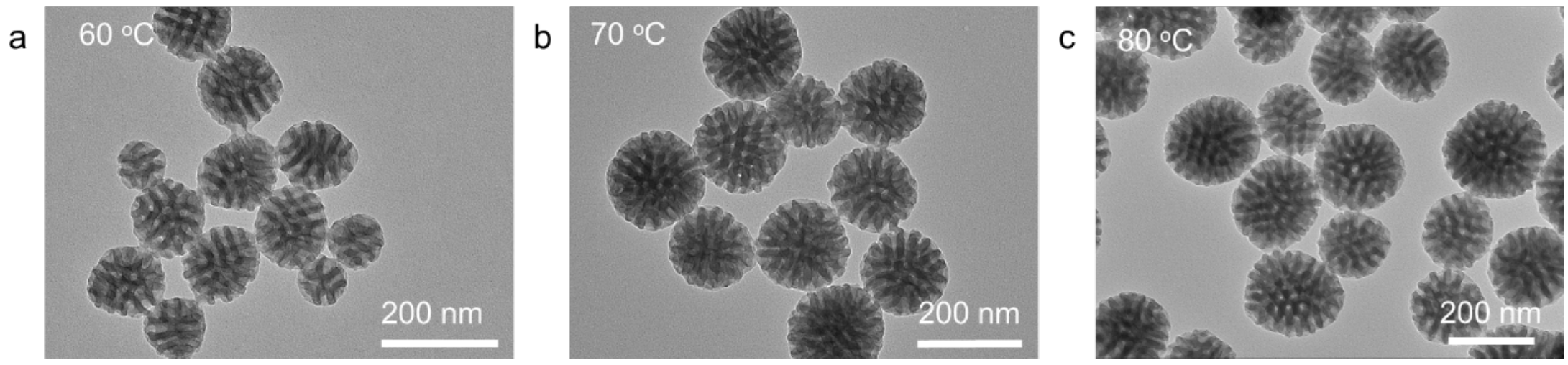

Figure S13. TEM images of mDSR prepared by the ionization modulation approach at different temperature.

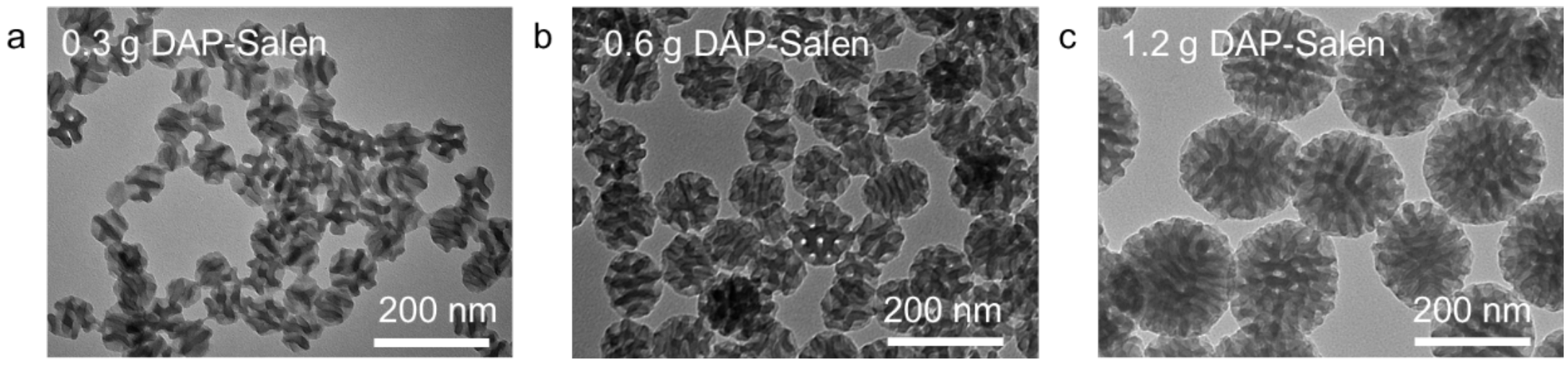

Figure S14. TEM images of mDSR prepared by the ionization modulation approach with different amounts of precursors.

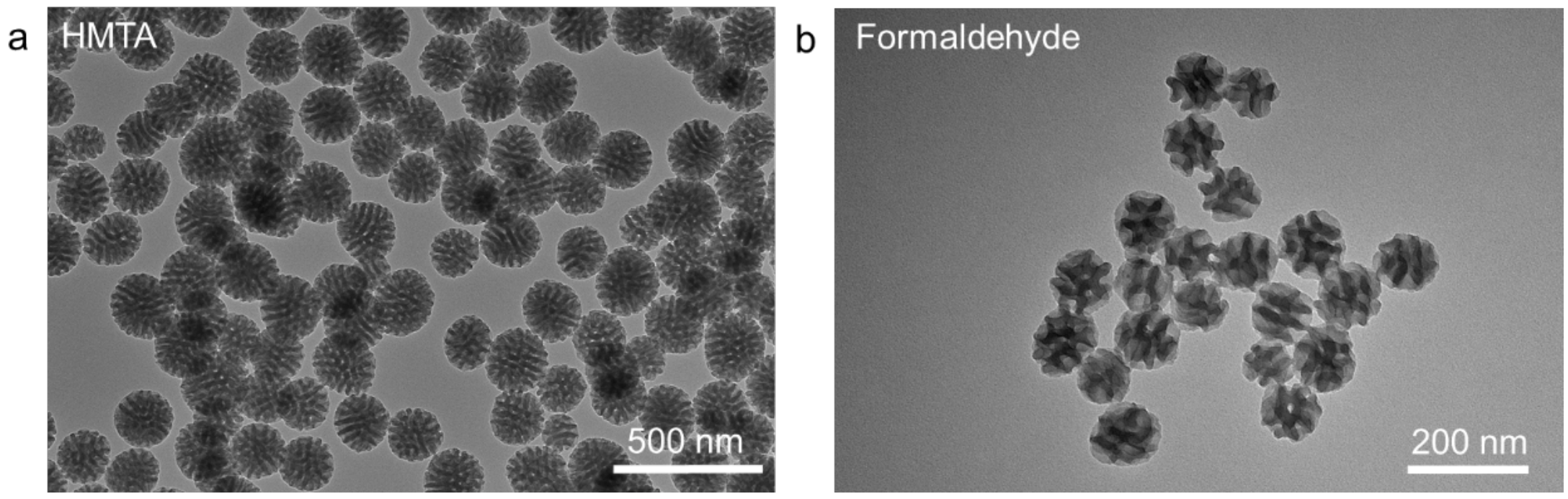

Figure S15. TEM images of mDSR prepared by the ionization modulation approach with different crosslinkers.

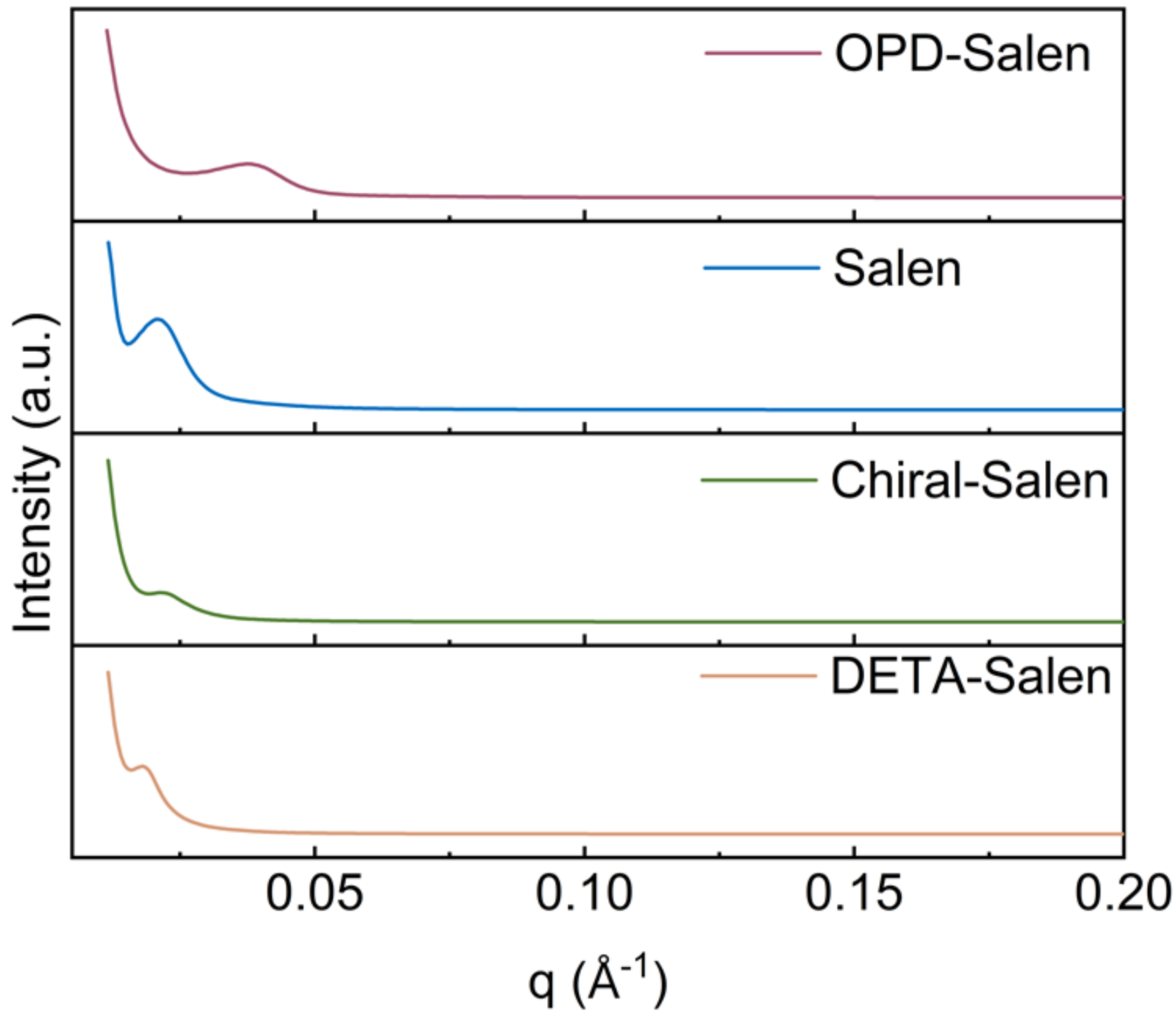

Figure S16. SAXS patterns of mesoporous polymer synthesized from different Salen/Salen-type ligands using the ionization regulating self-assembly strategy.

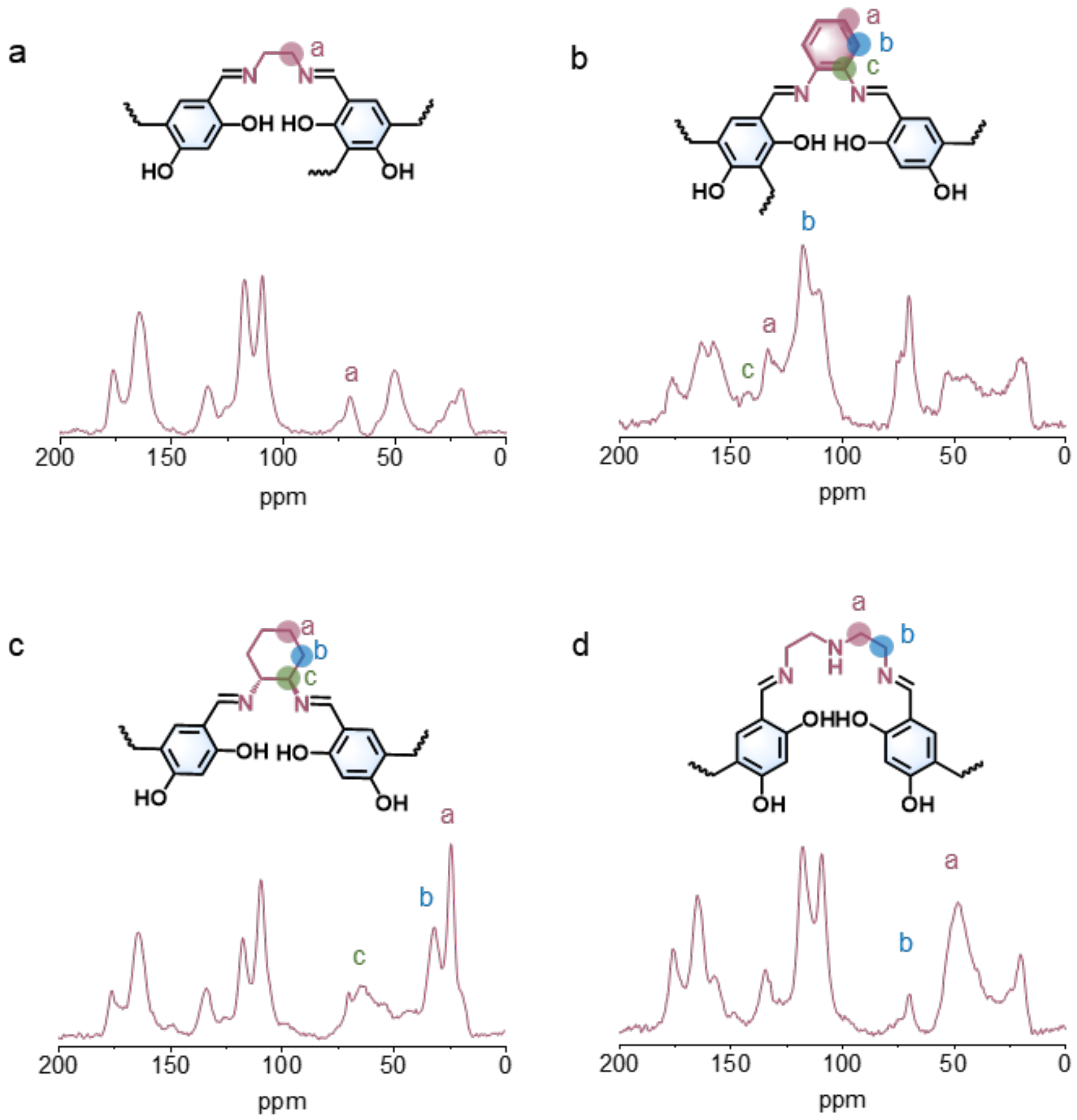

Figure S17. ^{13}C CP-MAS NMR spectra of mesoporous polymer synthesized from different Salen/Salen-type ligands using the ionization regulating self-assembly strategy.

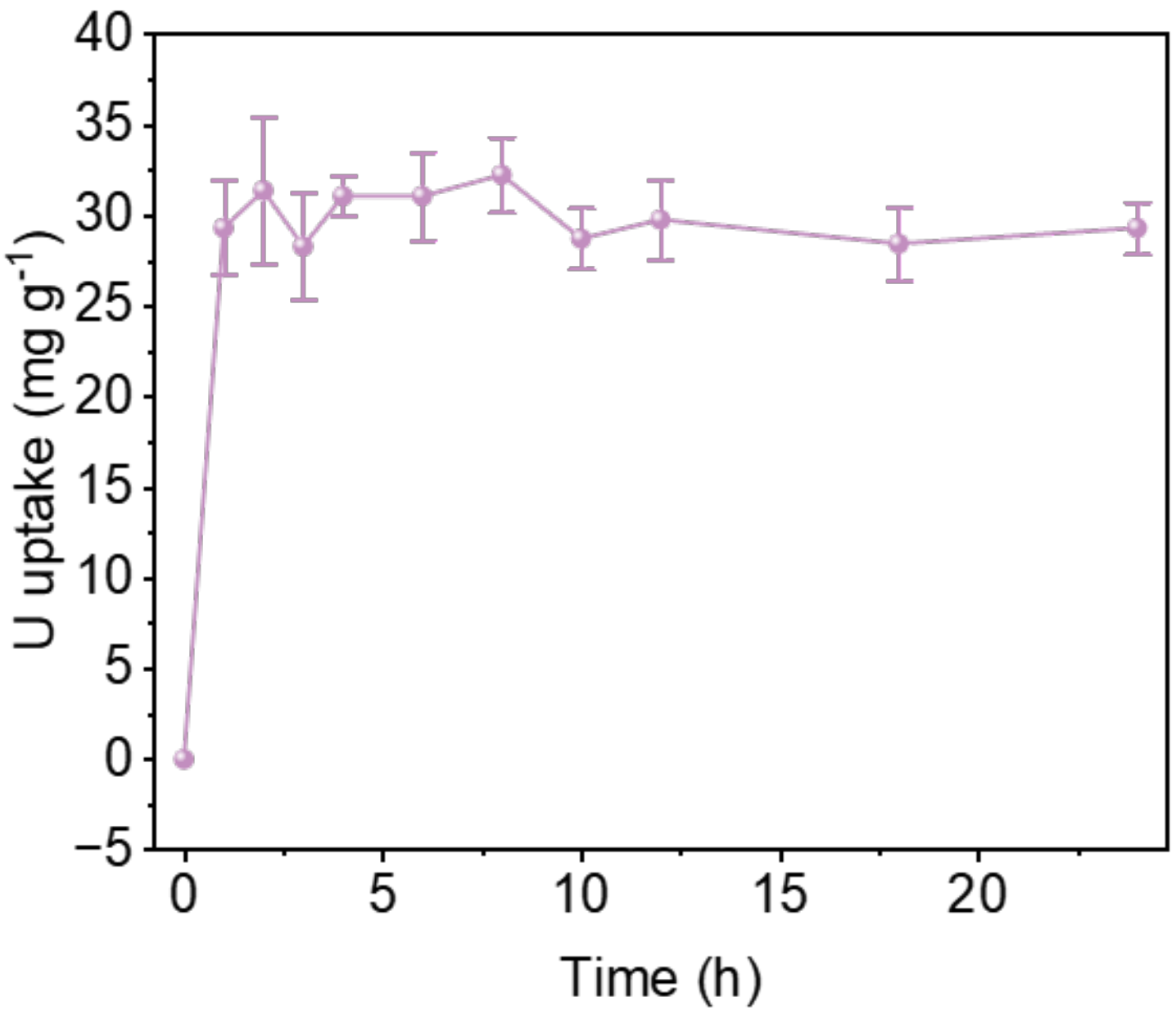

Figure S18. Uranium extraction capacity of mDSR at an initial U(VI) concentration of 10 ppm (pH = 6) in the dark.

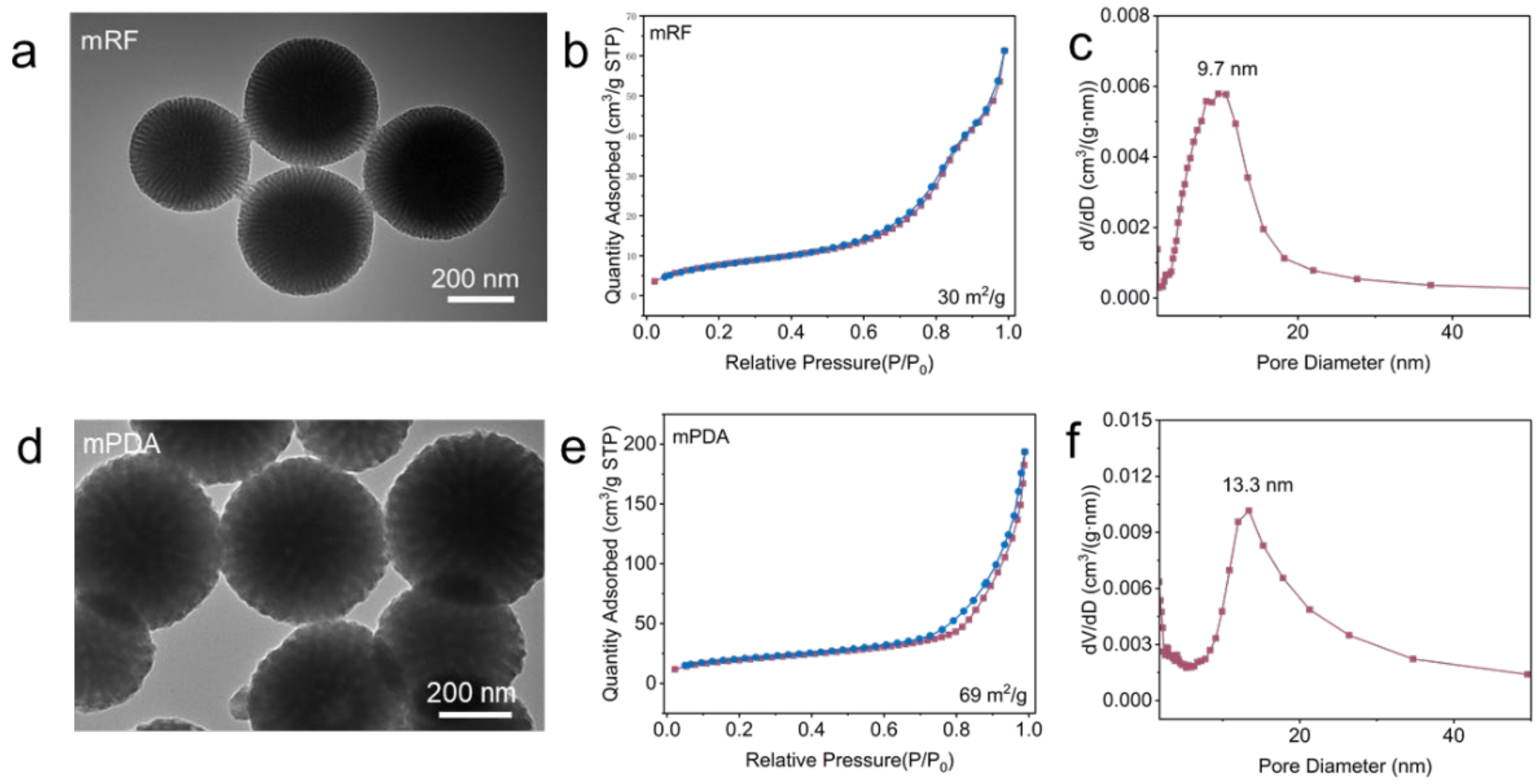

Figure S19. TEM images, N_2 sorption isotherms and corresponding pore-size distributions of (a-c) mRF and (d-f) mPDA.

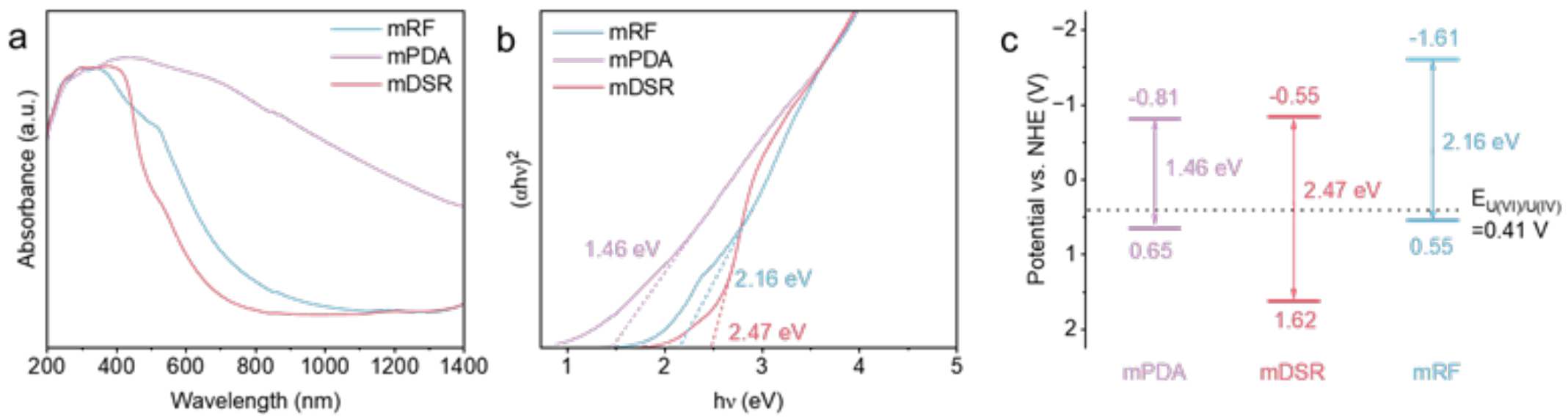

Figure S20. (a) UV-Vis diffuse reflectance spectra (b) Band gap patterns and (c) Energy band diagrams of mDSR, mPDA and mRF.

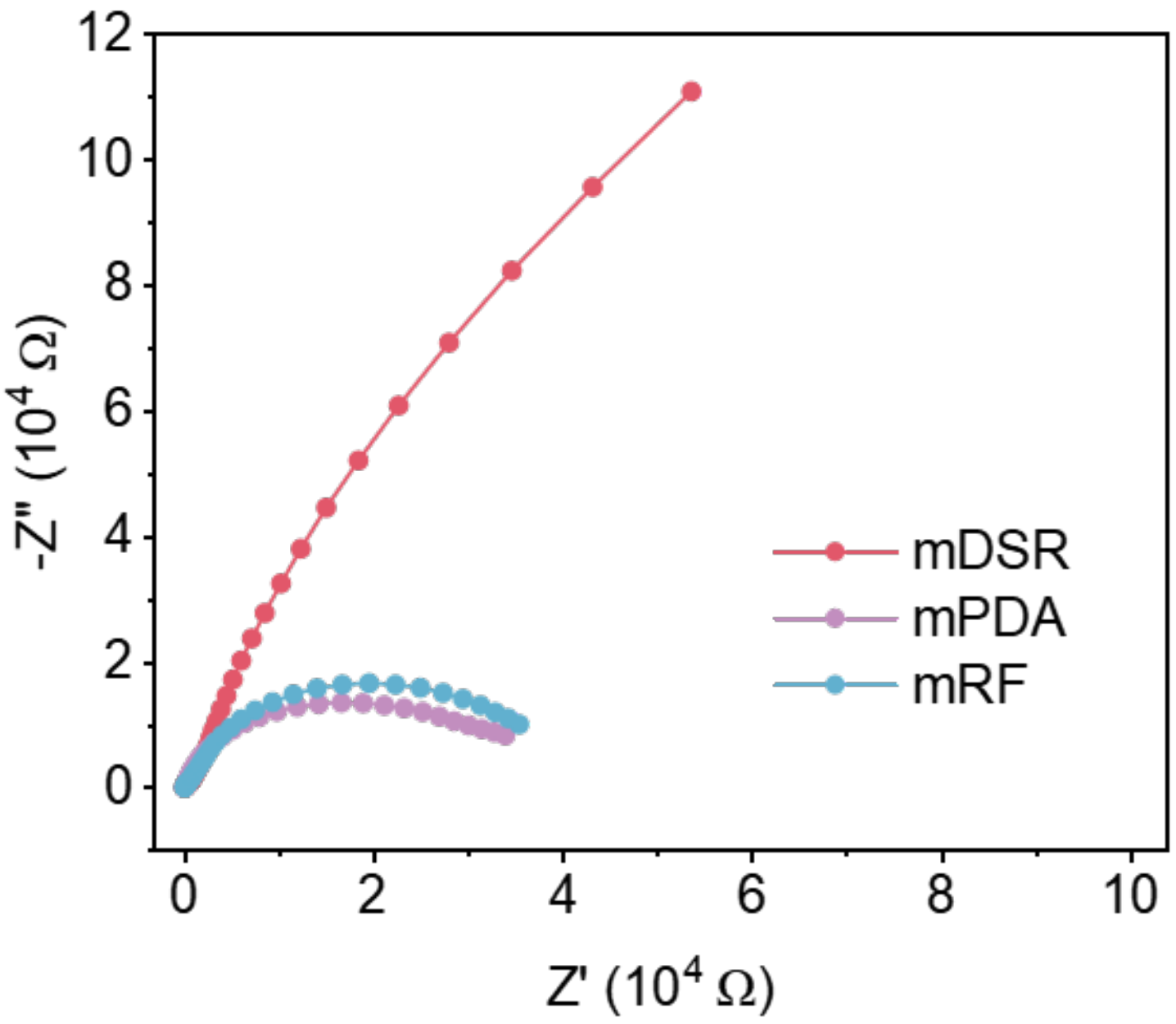

Figure S21. Electrochemical impedance spectroscopy curves of mDSR, mPDA, mRF.

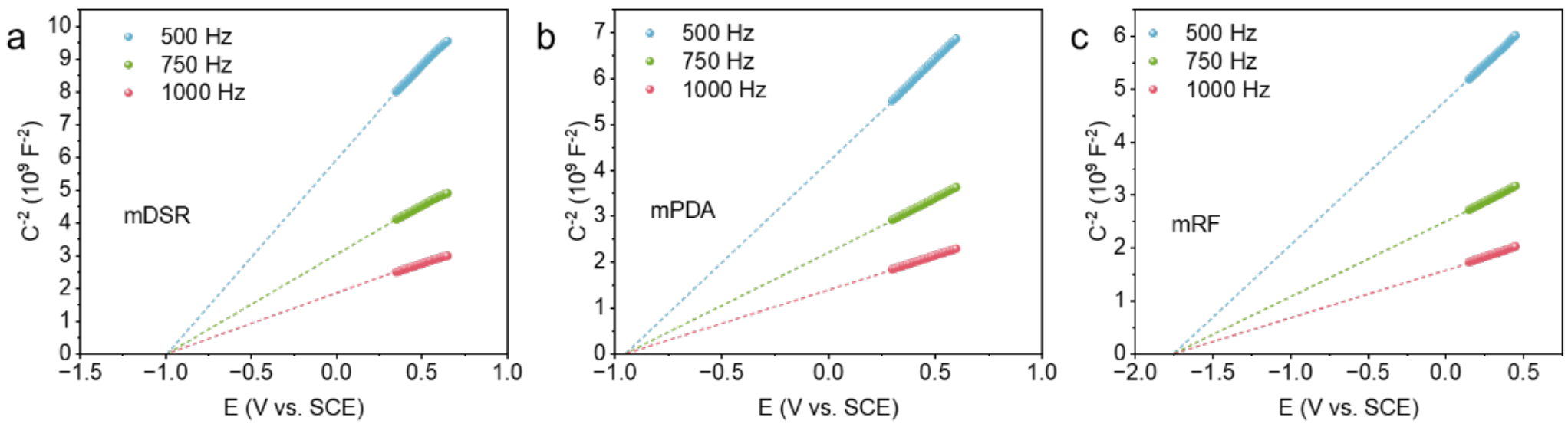

Figure S22. Mott–Schottky plots of (a) mDSR, (b) mPDA, (c) mRF. The samples were measured under 5 mV with the frequency of 500, 750, and 1000 Hz.

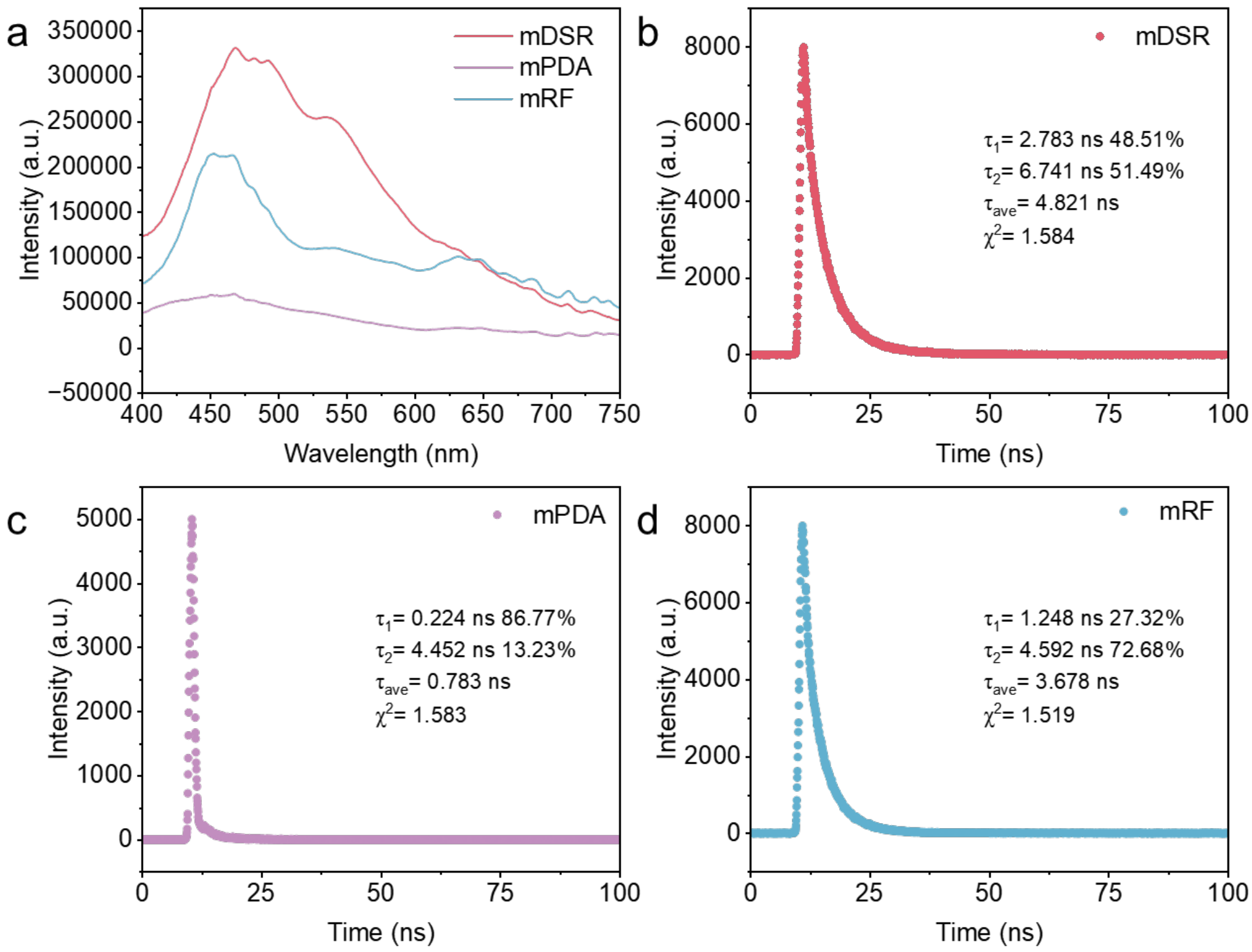

Figure S23. (a) Photoluminescence spectra of mDSR, mPDA, mRF under 375 nm excitation. (b-d) Time-resolved photoluminescence attenuation spectra were collected for mDSR, mPDA, mRF after excitation with 375 nm light.The τ_1 and τ_2 decay times correspond to radiative recombination of free electrons and holes and non-radiative energy transfer processes, respectively. The τ_{ave} is the average fluorescence lifetime calculated from τ_1 and τ_2.

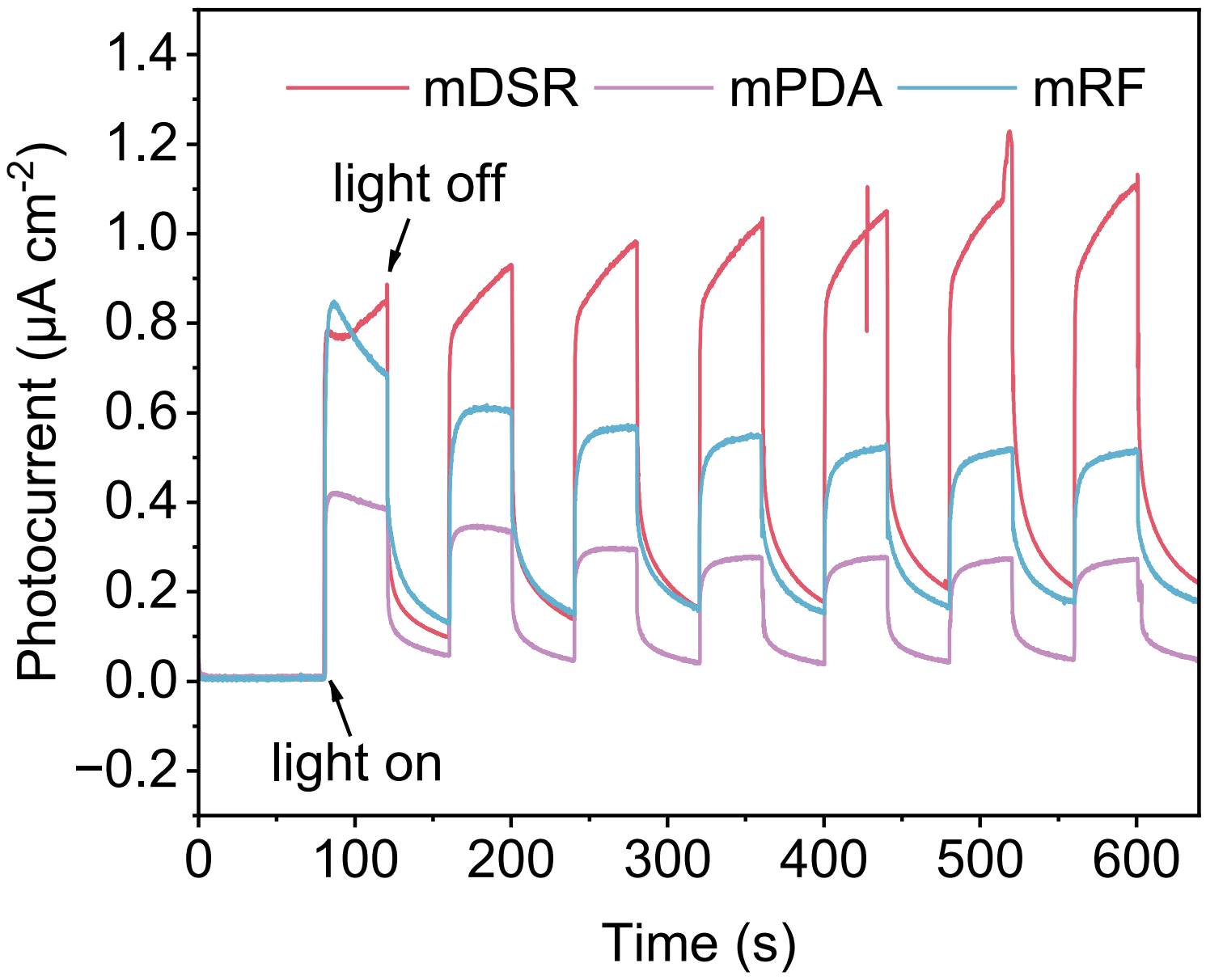

Figure S24. Transient photocurrent responses of mDSR, mPDA, and mRF

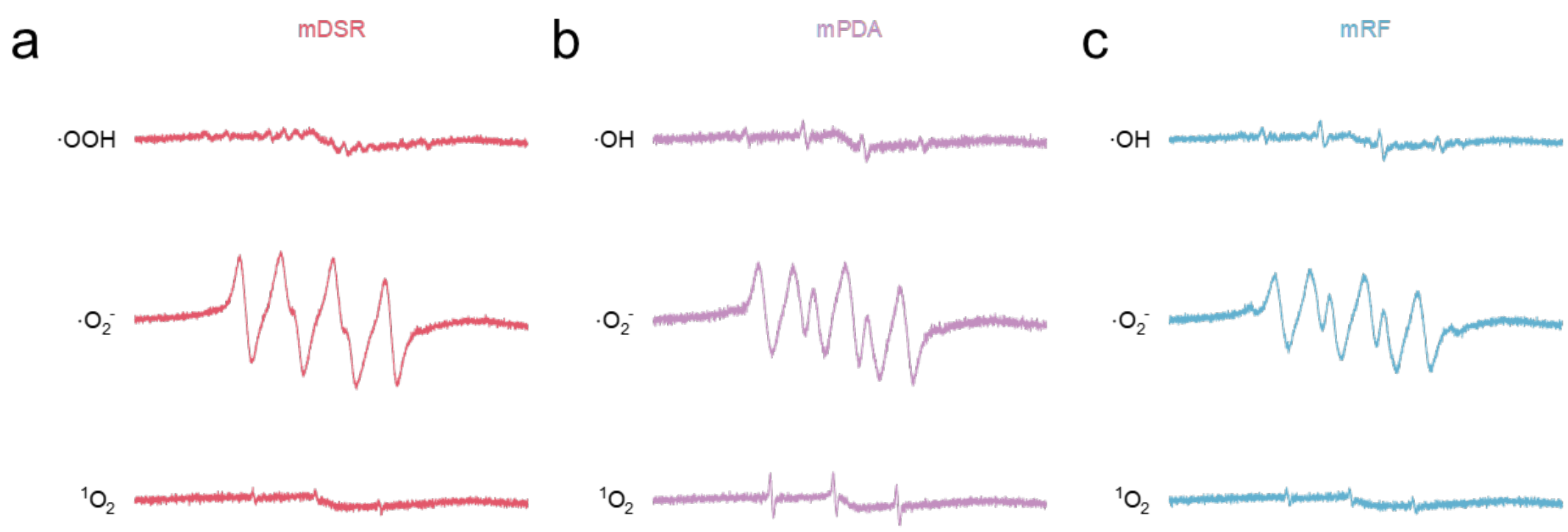

Figure S25. Reactive oxygen species generated by (a) mDSR, (b) mPDA, (c) mRF.

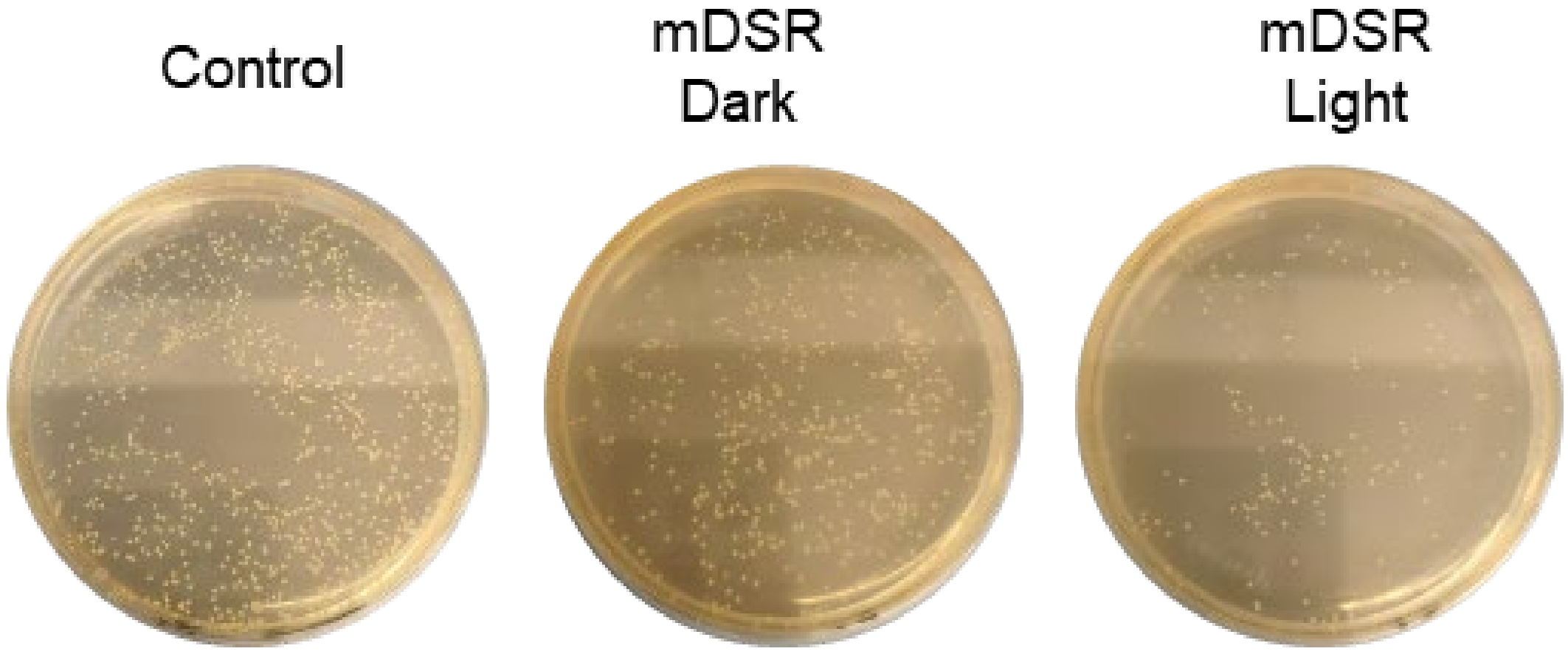

Figure S26. Antibacterial activity of mDSR, the bacterial strain used was methicillin-resistant Staphylococcus aureus (MRSA) ATCC 33591.

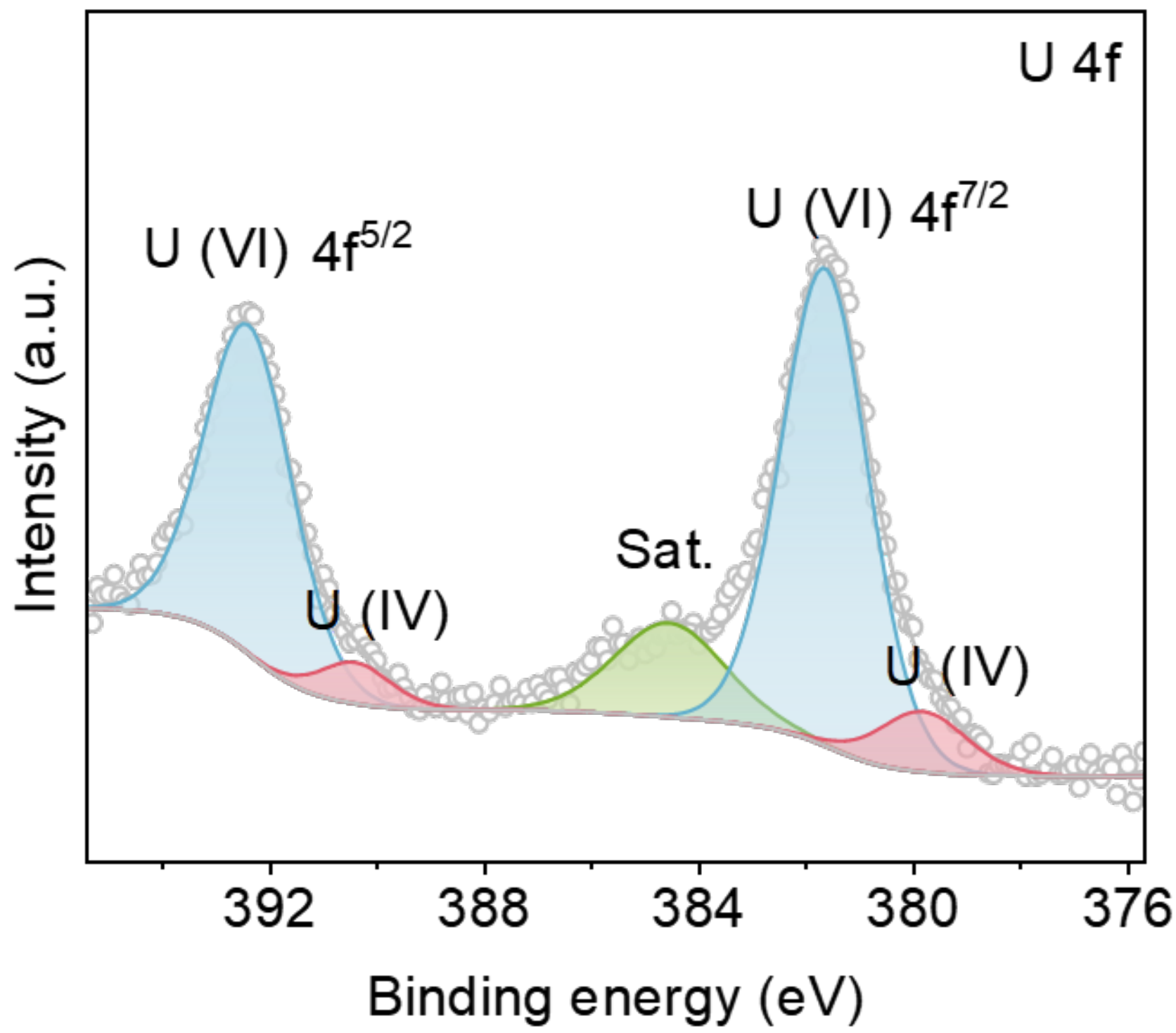

Figure S27. High-resolution U 4f XPS spectrum of mDSR after uranium extraction.

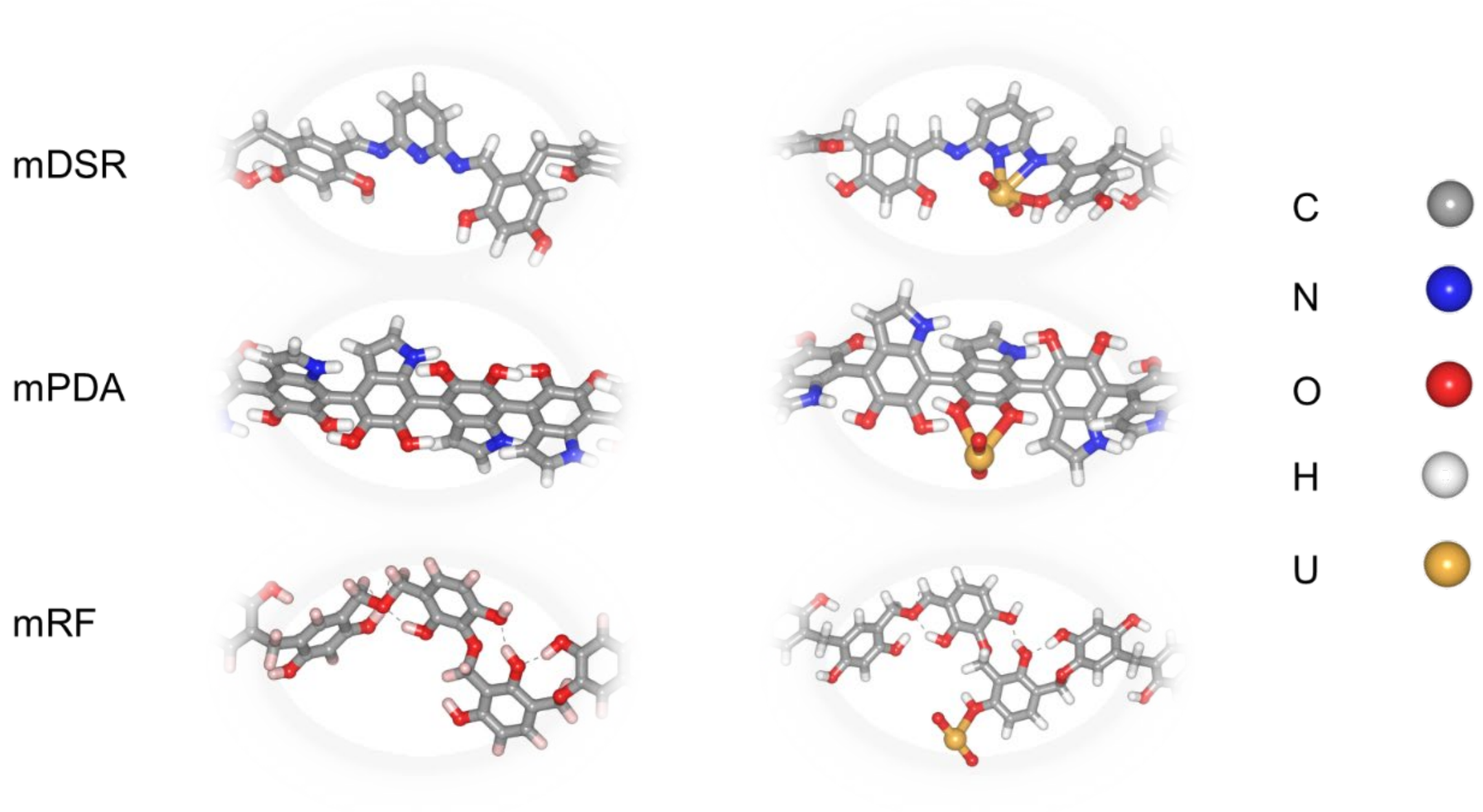

Figure S28. The optimized structures of mDSR, mPDA, and mRF polymer fragments and $*UO_2$ adsorption models on mDSR, mPDA and mRF.

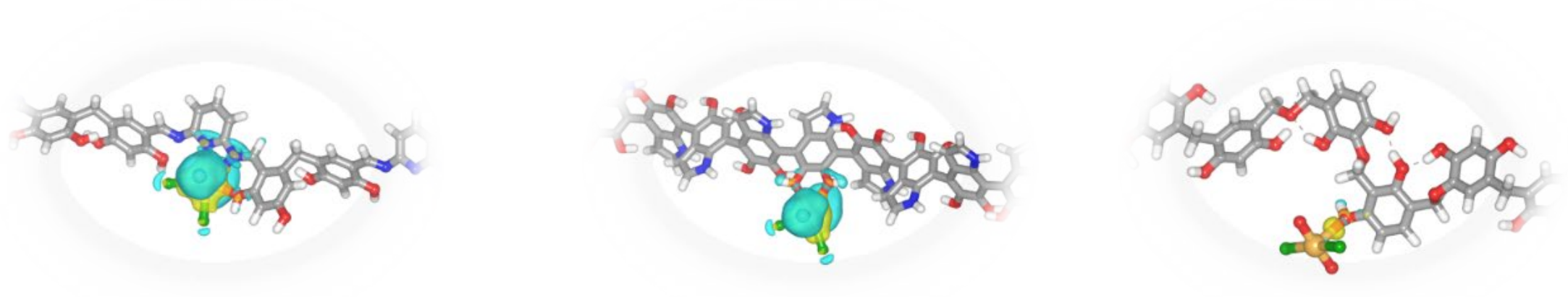

Figure S29. Electron charge difference analysis of $*UO_2^{2+}$ absorbed on the mDSR, mPDA and mRF.

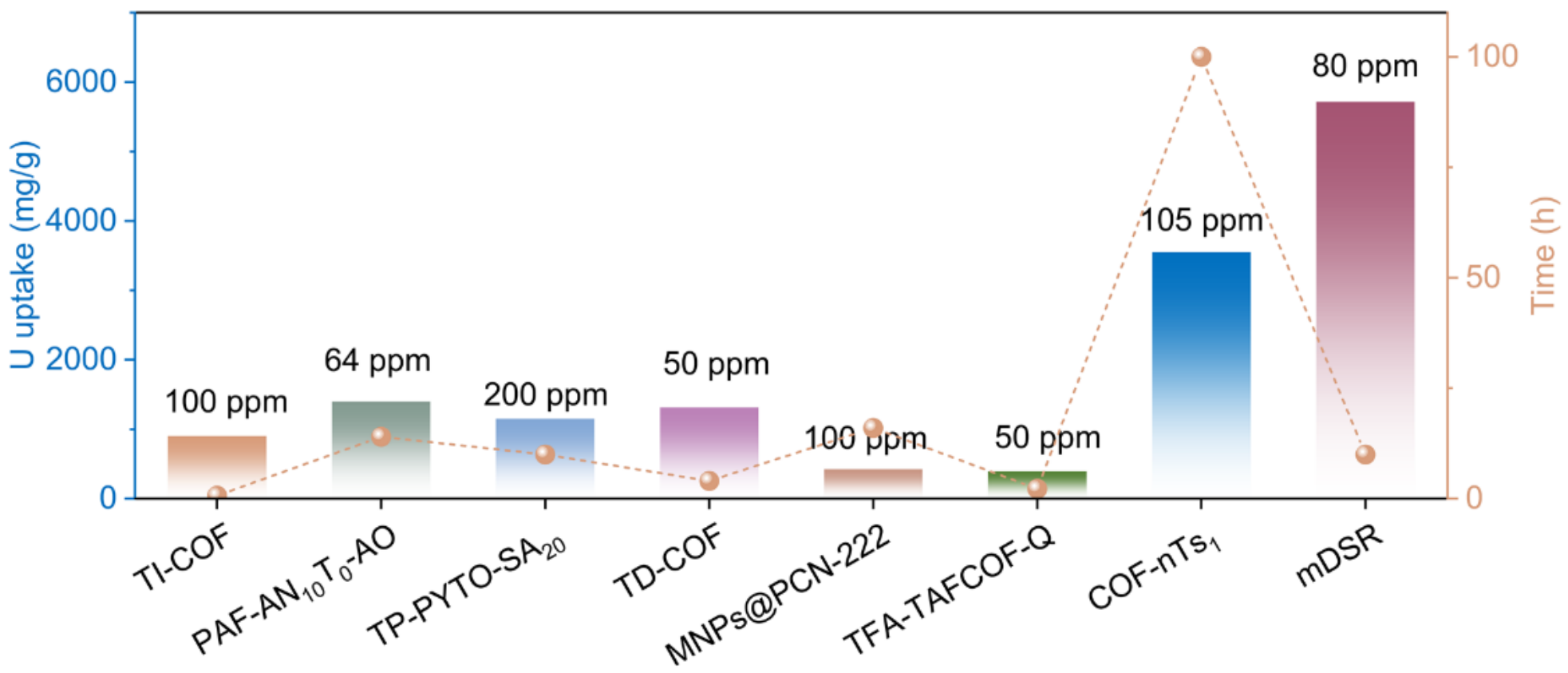

Figure S30. Comparison of the uranium extraction capacity of mDSR with previously reported materials under high uranium concentrations.

Tables.

Table S1. Fractional atomic coordinates for the unit cell of *UO_2^{2+} absorbed on the mDSR.

1	C	4	3.61252	-0.38748
2	C	4	3.887043	-0.112957
3	C	4	3.519985	-0.480015
4	C	4	4.00048	0.00048
5	C	4	4.020711	0.020711
6	C	4	4.004231	0.004231
7	C	4	4.033975	0.033975
8	C	4	3.972008	-0.027992
9	C	4	3.58545	-0.41455
10	C	4	3.948358	-0.051642
11	C	4	3.552429	-0.447571
12	C	4	3.958438	-0.041562
13	C	4	3.976947	-0.023053
14	C	4	3.387113	-0.612887
15	C	4	3.128144	-0.871856
16	C	4	3.163214	-0.836786
17	C	4	3.980536	-0.019464
18	C	4	4.077316	0.077316
19	C	4	4.063697	0.063697
20	C	4	3.341752	-0.658248
21	C	4	3.975191	-0.024809
22	C	4	3.579366	-0.420634
23	C	4	3.912374	-0.087626
24	C	4	3.541575	-0.458425
25	C	4	4.000403	0.000403
26	C	4	4.061865	0.061865
27	C	4	4.092242	0.092242
28	C	4	3.998208	-0.001792
29	C	4	3.54785	-0.45215
30	C	4	3.947833	-0.052167
31	C	4	3.476607	-0.523393
32	C	4	3.999593	-0.000407
33	C	4	4.09428	0.09428
34	C	4	3.335574	-0.664426
35	C	4	3.075652	-0.924348
36	C	4	3.164097	-0.835903
37	C	4	3.991927	-0.008073
38	C	4	3.979803	-0.020197

39	C	4	4.088229	0.088229
40	C	4	3.376824	-0.623176
41	O	6	7.19525	1.19525
42	O	6	7.130095	1.130095
43	O	6	7.203985	1.203985
44	O	6	7.150406	1.150406
45	O	6	7.153995	1.153995
46	O	6	7.162503	1.162503
47	O	6	7.198062	1.198062
48	O	6	7.163769	1.163769
49	O	6	6.882513	0.882513
50	O	6	6.88196	0.88196
51	N	5	6.183374	1.183374
52	N	5	6.210059	1.210059
53	N	5	6.200805	1.200805
54	N	5	6.189096	1.189096
55	N	5	6.209499	1.209499
56	N	5	6.247715	1.247715
57	H	1	0.876281	-0.123719
58	H	1	0.918954	-0.081046
59	H	1	0.916634	-0.083366
60	H	1	0.948018	-0.051982
61	H	1	0.367527	-0.632473
62	H	1	0.90516	-0.09484
63	H	1	0.925618	-0.074382
64	H	1	0.893405	-0.106595
65	H	1	0.932212	-0.067788
66	H	1	0.887971	-0.112029
67	H	1	0.904615	-0.095385
68	H	1	0.891173	-0.108827
69	H	1	0.366981	-0.633019
70	H	1	0.893288	-0.106712
71	H	1	0.870696	-0.129304
72	H	1	0.897381	-0.102619
73	H	1	0.922588	-0.077412
74	H	1	0.893157	-0.106843
75	H	1	0.938383	-0.061617
76	H	1	0.872414	-0.127586
77	H	1	0.856624	-0.143376
78	H	1	0.935872	-0.064128
79	H	1	0.919648	-0.080352
80	H	1	0.916496	-0.083504
81	H	1	0.370279	-0.629721

82	H	1	0.32465	-0.67535
83	H	1	0.316616	-0.683384
84	H	1	0.321111	-0.678889
85	H	1	0.298436	-0.701564
86	H	1	0.297433	-0.702567
87	U	14	11.255263	-2.744737
88	Cl	7	7.651082	0.651082
89	Cl	7	7.69711	0.69711

Table S2. Fractional atomic coordinates for the unit cell of *UO_2^{2+} absorbed on the mPDA.

1	C	4	3.987146	-0.012854
2	C	4	3.538889	-0.461111
3	C	4	3.492348	-0.507652
4	C	4	3.948426	-0.051574
5	C	4	3.643253	-0.356747
6	C	4	4.001609	0.001609
7	C	4	4.06682	0.06682
8	C	4	3.736668	-0.263332
9	C	4	3.994586	-0.005414
10	C	4	3.505284	-0.494716
11	C	4	3.462724	-0.537276
12	C	4	4.057023	0.057023
13	C	4	3.666873	-0.333127
14	C	4	4.027865	0.027865
15	C	4	4.045322	0.045322
16	C	4	3.644394	-0.355606
17	C	4	3.970969	-0.029031
18	C	4	3.530279	-0.469721
19	C	4	3.544849	-0.455151
20	C	4	4.001896	0.001896
21	C	4	3.614892	-0.385108
22	C	4	3.983759	-0.016241
23	C	4	4.046773	0.046773
24	C	4	3.6808	-0.3192
25	C	4	4.010055	0.010055
26	C	4	3.531681	-0.468319
27	C	4	3.576159	-0.423841
28	C	4	3.964324	-0.035676
29	C	4	3.609858	-0.390142
30	C	4	3.979433	-0.020567
31	C	4	4.022132	0.022132
32	C	4	3.724024	-0.275976
33	N	5	6.275389	1.275389
34	N	5	6.248462	1.248462
35	N	5	6.291994	1.291994
36	N	5	6.304742	1.304742
37	O	6	7.147591	1.147591
38	O	6	7.132571	1.132571
39	O	6	7.129529	1.129529
40	O	6	7.181587	1.181587
41	O	6	7.150447	1.150447

42	O	6	7.18145	1.18145
43	O	6	7.179503	1.179503
44	O	6	7.225065	1.225065
45	H	1	0.893445	-0.106555
46	H	1	0.84271	-0.15729
47	H	1	0.47447	-0.52553
48	H	1	0.366883	-0.633117
49	H	1	0.380955	-0.619045
50	H	1	0.929671	-0.070329
51	H	1	0.914989	-0.085011
52	H	1	0.392053	-0.607947
53	H	1	0.327721	-0.672279
54	H	1	0.905248	-0.094752
55	H	1	0.885845	-0.114155
56	H	1	0.459081	-0.540919
57	H	1	0.354744	-0.645256
58	H	1	0.336318	-0.663682
59	H	1	0.882092	-0.117908
60	H	1	0.854756	-0.145244
61	H	1	0.45898	-0.54102
62	H	1	0.308914	-0.691086
63	H	1	0.254347	-0.745653
64	H	1	0.490474	-0.509526
65	U	14	11.168322	-2.831678
66	O	6	6.867953	0.867953
67	O	6	6.873151	0.873151
68	Cl	7	7.667037	0.667037
69	Cl	7	7.650397	0.650397

Table S3. Fractional atomic coordinates for the unit cell of *UO_2^{2+} absorbed on the mRF.

1	C	4	3.484097	-0.515903
2	C	4	3.515087	-0.484913
3	C	4	4.076597	0.076597
4	C	4	4.009509	0.009509
5	C	4	3.507376	-0.492624
6	C	4	4.047632	0.047632
7	C	4	4.028369	0.028369
8	C	4	4.030067	0.030067
9	C	4	3.517525	-0.482475
10	C	4	3.992136	-0.007864
11	C	4	3.547392	-0.452608
12	C	4	4.015084	0.015084
13	C	4	4.05119	0.05119
14	C	4	3.617241	-0.382759
15	C	4	3.605728	-0.394272
16	C	4	3.952617	-0.047383
17	C	4	3.539632	-0.460368
18	C	4	3.439179	-0.560821
19	C	4	3.56788	-0.43212
20	C	4	4.010536	0.010536
21	C	4	4.096736	0.096736
22	C	4	3.63813	-0.36187
23	C	4	3.993135	-0.006865
24	C	4	3.512384	-0.487616
25	C	4	3.95757	-0.04243
26	C	4	4.035862	0.035862
27	C	4	4.083512	0.083512
28	C	4	3.572087	-0.427913
29	C	4	3.587884	-0.412116
30	O	6	7.170771	1.170771
31	O	6	7.186008	1.186008
32	O	6	7.038604	1.038604
33	O	6	7.169979	1.169979
34	O	6	7.170375	1.170375
35	O	6	7.19692	1.19692
36	O	6	7.195089	1.195089
37	O	6	7.044886	1.044886
38	O	6	7.063262	1.063262
39	O	6	7.188172	1.188172
40	O	6	7.211779	1.211779
41	H	1	0.937281	-0.062719

42	H	1	0.875131	-0.124869
43	H	1	0.931788	-0.068212
44	H	1	0.940829	-0.059171
45	H	1	0.884129	-0.115871
46	H	1	0.960641	-0.039359
47	H	1	0.382371	-0.617629
48	H	1	0.350829	-0.649171
49	H	1	0.924213	-0.075787
50	H	1	0.940356	-0.059644
51	H	1	0.935025	-0.064975
52	H	1	0.946779	-0.053221
53	H	1	0.338471	-0.661529
54	H	1	0.350316	-0.649684
55	H	1	0.906389	-0.093611
56	H	1	0.900579	-0.099421
57	H	1	0.330948	-0.669052
58	H	1	0.301493	-0.698507
59	H	1	0.908523	-0.091477
60	H	1	0.915377	-0.084623
61	H	1	0.892779	-0.107221
62	H	1	0.876252	-0.123748
63	H	1	0.942388	-0.057612
64	H	1	0.938041	-0.061959
65	H	1	0.346206	-0.653794
66	H	1	0.273314	-0.726686
67	U	14	11.070875	-2.929125
68	O	6	6.869535	0.869535
69	O	6	6.864122	0.864122
70	Cl	7	7.661416	0.661416
71	Cl	7	7.635583	0.635583

Table S4. Summary of adsorbents used for photocatalytic extraction of uranium from an aqueous solution of uranyl(VI) at similar conditions.

Adsorbent	Tesr time (h)	U(VI) concentrations (ppm)	Uranium capacity (mg g^{-1})	Ref.
mDSR	1	10	198	This work
mDSR	10	10	867	This work
ORP-CN	2	2	58.17	[10]
COF-4	24	24	182	[11]
PAF-$AN_{10}T_0$-AO	14	14	135.74	[12]
SCN-C_3N_4	8	8	290.7	[13]
BB-COP-AO	16	16	99.65	[14]
BTT-COF-AO	16	16	786	[15]
N_3-COF_{60}	168	10	1270	[4]
mDSR	10	60	4136	This work
mDSR	10	80	5715	This work
TI-COF	0.67	100	902	[16]
PAF-$AN_{10}T_0$-AO	14	64	1398.42	[12]
TP-PYTO-SA_{20}	10	200	1151.65	[17]
TD-COF	4	50	1312.7	[18]
MNPs@PCN-222	16	100	423.8	[19]
TFA-TAFCOF-Q	2.25	50	391.6	[20]
COF-TpTapb-COOH	252	500	7078.8	[21]
COF-nTs_1	100	105	3548.1	[22]
N_3-COF_{60}	480	100	10900	[4]

References

[1] Milosevic, M. D.; Marinkovic, A. D.; Petrovic, P.; Klaus, A.; Nikolic, M. G.; Prlainovic, N. Z.; Cvijetic, I. N. Synthesis, characterization and SAR studies of bis(imino)pyridines as antioxidants, acetylcholinesterase inhibitors and antimicrobial agents. *Bioorg. Chem.* **2020**, *102*, 104073.

[2] Peng, L.; Peng, H. R.; Li, W.; Zhao, D. Y. Monomicellar assembly to synthesize structured and functional mesoporous carbonaceous nanomaterials. *Nat. Protoc.* **2023**, *18* (4), 1155-1178.

[3] Makula, P.; Pacia, M.; Macyk, W. How to correctly determine the band gap energy of modified semiconductor photocatalysts based on UV-Vis spectra. *J. Phys. Chem. Lett.* **2018**, *9* (23), 6814-6817.

[4] Ma, X. J.; Meihaus, K. R.; Yang, Y. J.; Zheng, Y.; Cui, F. C.; Li, J. X.; Zhao, Y. Q.; Jiang, B.; Yuan, Y.; Long, J. R.; et al. Photocatalytic extraction of uranium from seawater using covalent organic framework nanowires. *J. Am. Chem. Soc.* **2024**, *146* (33), 23566-23573.

[5] Guo, L. C.; Liu, Y. X.; Jia, Y. Z.; Huang, Z. C.; Gong, L. L.; Xie, X. Q.; Luo, F. Control on the stacking mode in a sulfonic covalent organic framework enabling a precise uranyl-identified pocket for ultrahigh-capacity uranium extraction from seawater. *J. Am. Chem. Soc.* **2025**, *147* (34), 31340-31348.

[6] Guo, L. C.; Yang, Y. T.; Gong, L. L.; Zhang, Q. Y.; Liao, Y. X.; Hua, R.; Yu, Z. W.; Luo, F. Innovative conversion strategy for wastewater with one-pot uranium extraction and valuable chemical production by a smart COF photocatalyst. *Adv. Funct. Mater.* **2024**, *34* (29), 2400588.

[7] Liang, B. C.; Wu, H. N.; Chen, L. J.; Zhao, X. Y.; Gao, Y.; Liu, Y. L.; Dai, Z. R. Polydopamine-mediated photocatalytic uranium removal under ambient conditions: Performance and mechanism. *Appl. Surf. Sci.* **2026**, *723*, 165619.

[8] Mathew, K.; Kolluru, V. S. C.; Mula, S.; Steinmann, S. N.; Hennig, R. G. Implicit self-consistent electrolyte model in plane-wave density-functional theory. *J. Chem. Phys.* **2019**, *151*, 234101.

[9] Mathew, K.; Sundararaman, R.; Letchworth-Weaver, K.; Arias, T. A.; Hennig, R. G. Implicit solvation model for density-functional study of nanocrystal surfaces and reaction pathways. *J. Chem. Phys.* **2014**, *140*, 084106.

[10] Yang, Y. H.; Zhang, L. X.; Zhang, L. Y.; Qing, Q.; Liu, S.; Wang, Z.; Xiong, X.-G.; Wang, X. K.; Lu, Y. X. Directing intermediates in photocatalytic H_2O_2 production to boost uranium extraction from seawater. *Small* **2026**, *22* (13), e09062.
[11] Chen, Z. S.; Wang, J. Y.; Hao, M. J.; Xie, Y. H.; Liu, X. L.; Yang, H.; Waterhouse, G. I. N.; Wang, X. K.; Ma, S. Q. Tuning excited state electronic structure and charge transport in covalent organic frameworks for enhanced photocatalytic performance. *Nat. Commun.* **2023**, *14* (1), 1106.
[12] Ma, Y.; Liu, L.; Zhao, S. L.; Xu, X. Z.; Wang, Y.; Zhang, Y. L.; Rong, H. Z.; Yuan, Y. H.; Wang, N. Molecular engineering of multivariate porous aromatic frameworks for recovery of dispersed uranium resources. *Adv. Funct. Mater.* **2024**, *34* (52), 2410778.
[13] Wang, P.; Hu, E. M.; Wu, L. Z.; He, J. H.; Wang, Y. H.; Ou, Z. H.; Yang, F.; Chen, T.; Zhu, W. K. Tailoring the electronic microenvironment of polymeric carbon nitride to accelerate reduction crystallization kinetics of uranium extraction from seawater. *Appl. Catal. B Environ* **2025**, *371*, 125266.
[14] Tian, X.; Li, J.; Cao, X.; Zhang, J.; Huang, R.; Lin, P.; Wang, N.; Yuan, Y. Conjugated organic polymer enables external-sacrificial-agent-free photocatalytic uranium extraction with efficiency boosted by oxygen pre-activation. *Sci. Bull. (Beijing)* **2026**, *71* (7), 1686-1694.
[15] Ma, Y.; Gan, C.; Liu, L.; Pan, J. J.; Rong, H. Z.; Guo, Y.; Yuan, Y. H.; Wang, N. Polarization-π strategy for modulating excited-state band structures of covalent organic frameworks to enhance photocatalytic uranium extraction. *Adv. Funct. Mater.* **2025**, e23125.
[16] Zhong, L. Z.; Feng, X. F.; Zhang, Q. Y.; Xie, X. Q.; Luo, F. An imidazole-based covalent-organic framework enabling a super-efficiency in sunlight-driven uranium extraction from seawater. *Chem. Sci.* **2024**, *15* (28), 10882-10891.
[17] Yao, W. K.; Zhou, G. B.; Liu, T.; Yuan, Y. H.; Wang, H.; Wang, N. Defect engineering activates pyrene-based covalent organic frameworks for efficient photocatalytic uranium capture. *Adv. Sci.* **2025**, *13* (11), e18948.
[18] He, Y. X.; Yu, F. T.; Gui, J. L.; Zhang, Z. W.; Zhang, C. R.; Liang, R. P.; Qiu, J. D. Breaking the exciton bottleneck in 1D COFs via tailored active-sites microenvironment enables high-capacity uranium photocatalytic. *Adv. Funct. Mater.* **2026**, *36* (11), e17374.
[19] Yang, Y. A.; Li, S. H.; Xue, S. L.; Chang, S. R.; Pan, J. M.; Xing, W. H. Functionalized porphyrin MOF with embedded magnetic core for rapid and recyclable photocatalytic uranium extraction. *Small* **2025**, *21* (51), e09754.

[20] Liu, C.; Wang, Y. X.; Dong, Z. M.; Zhang, Z. B.; Cao, X. H.; Zhai, Y. M.; Liu, Y. H. Covalent organic frameworks for enhanced photocatalytic extraction of uranium via the modulation of charge transfer pathways. *Chem. Eng. J.* **2025**, *514*, 163078.
[21] Hussain, M.; Kim, B.; Ullah, I.; Devarayapalli, K. C.; Kumaravel, S.; Lee, D. S. Constructing covalent organic frameworks via a multicomponent Doebner reaction for enhanced hydrogen peroxide-driven uranium removal. *Chem. Eng. J.* **2025**, *521*, 166567.
[22] Ahmad, M.; Mao, X. M.; Zhao, K. H.; Naik, M. U. D.; Tariq, M. R.; Khan, I.; Zhang, B. L. Morphology controlled covalent organic framework nano-trap for synergetic uranium adsorption and photoreduction. *Small* **2025**, *21* (22), 2501578.
